# Single-cell and bulk tissue sequencing unravels the heterogeneity of synovial microenvironment in arthrofibrosis

**DOI:** 10.1016/j.isci.2023.107379

**Published:** 2023-07-13

**Authors:** Xi Chen, Lihua Gong, Cheng Li, Siyuan Wang, Ziyuan Wang, Ming Chu, Yixin Zhou

**Affiliations:** 1 Department of Adult Joint Reconstructive Surgery, Beijing Jishuitan Hospital, Capital Medical University, 31 East Xinjiekou Street, Beijing 100035, China; 2 Department of Immunology, School of Basic Medical Sciences, Peking University, NHC Key Laboratory of Medical Immunology (Peking University), Beijing, China; 3 Department of Pathology, National Cancer Center/National Clinical Research Center for Cancer/Cancer Hospital, Chinese Academy of Medical Sciences and Peking Union Medical College, Beijing 100021, China

**Keywords:** Fibrosis, Molecular biology experimental approach, Immune system disorder, Transcriptomics

## Abstract

Arthrofibrosis (AF) is a debilitating complication that occurs after trauma or surgery, leading to functional impairment and surgical failures worldwide. This study aimed to uncover the underlying mechanism of AF. A total of 141 patients were enrolled, and synovial samples were collected from both patients and animal models at different time points. Single-cell RNA-sequencing (scRNA-seq) and bulk tissue RNA sequencing (bulk-seq) were employed to profile the distinct synovial microenvironment. This study revealed changes in cell proportions during AF pathogenesis and identified Engrailed-1 (EN1) as a key transcription factor strongly associated with disease severity and clinical prognosis. Additionally, the researchers discovered a specific type of synovial fibroblast called DKK3-SLF, which played a critical role in driving AF development. These findings shed light on the composition and heterogeneity of the synovial microenvironment in AF, offering potential avenues for identifying therapeutic targets and developing clinical treatments for AF and other fibrotic diseases.

## Highlights

• This study unveils synovial microenvironment landscape of AF using scRNA-seq
• EN1 identified as key transcription factor linked to AF severity
• DKK3-SLF revealed as crucial pathological cell type driving AF development
• An online arthrofibrosis database were established within these data

## Graphical abstract

**Figure:**
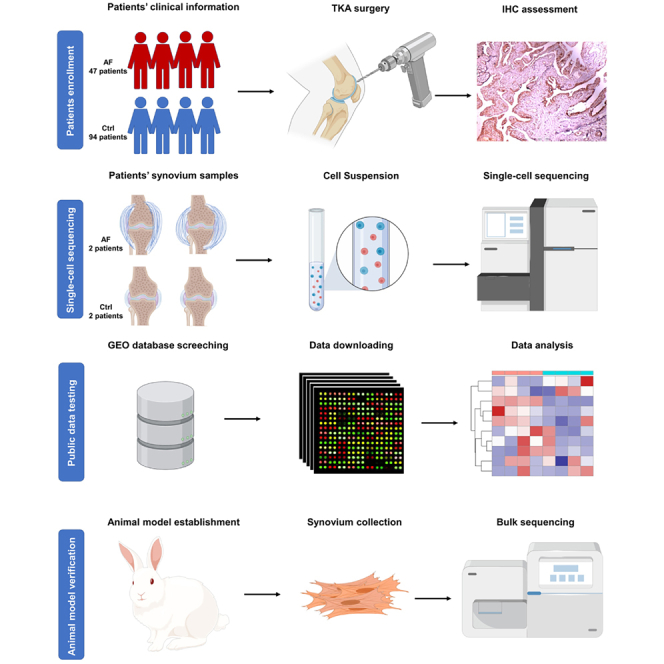

## Introduction

Arthrofibrosis (AF), also known as joint stiffness, is a well-known complication after surgery or trauma. It was previously described as a form of fibrotic disease with excessive collagen deposition and fibrous scar formation in the joint cavity.^1^ This pathological change permanently decreases the range of motion (ROM) of patients’ joints after surgery and causes severe restriction of daily activities in driving, walking, sitting, and even sleeping.^2^^,^^3^^,^^4^ Furthermore, AF is always accompanied with stubborn and unbearable pain which requires a secondary revision surgery.^5^ Along with the surge in orthopedic surgery volume worldwide, AF has become a heavy burden on both patients and many clinical practitioners.^6^^,^^7^^,^^8^^,^^9^

Unfortunately, there is neither an effective way to prevent AF in the early stage nor conservative treatment in the late stage. The major barriers are the limited understanding of AF cellular mechanisms operated within the synovial microenvironment.^2^ Recent findings from various laboratories, including our own, clearly suggest that modulation of the local microenvironment is the central determinant of fibrotic pathogenesis.^10^^,^^11^^,^^12^ The regulation of heterogeneous tissue resident synovial cells and complicated cell communication networks in synovial microenvironment are key to fibrosis initiation and maintenance. However, previous studies only involved a few cellular types, which constrains the understanding of AF. Therefore, elucidation of the landscape of the synovial microenvironment is warranted for AF pathophysiology exploration.

The rapid development of single-cell RNA-sequencing (scRNA-seq) technologies provides a solution for profiling thousands of cell type-specific transcriptomes and revealing the heterogeneity of cellular functionality in the synovial microenvironment.^13^^,^^14^ It is a powerful technology to outline changes in different cell expression patterns and related cell-cell interactions. Bulk tissue RNA sequencing (Bulk-seq) is also a widely used method for exploring genome-wide transcriptomic variations. It measures the sum of gene expression weighted by different cellular type proportions. Combined with scRNA-seq data, recent studies have developed several reliable deconvolution methods for cell type proportion estimation of bulk-seq data, which provide possibilities for integration analysis.^15^^,^^16^

Here, we developed an integration study pipeline for sequencing data from different AF synovia. AF patients were enrolled after both pathological and clinical confirmation. Bulk-seq and scRNA-seq from human synovial tissue and a time series of animal synovial tissue were performed in this study. Based on this integration analysis, we identified specific pathogenic cellular phenotypes and uncovered functional heterogeneity in the synovial microenvironment.

## Results

### Pathological analysis of arthrofibrosis synovium

This study was integrated research on the AF microenvironment with four different level sequencing datasets (Figure S1). A total of 141 patients (47 AF and 94 non-AF) who underwent revision total knee arthroplasty (TKA) surgery were involved in this study after being reviewed with inclusive and exclusive criteria. X-ray images and gross images both show the significant thickening of the joint synovium (Figures S2A and S2B). The ROM and hydroxyproline contents were also checked and documented blindly by at least 2 surgeons. The ROM of AF patients sharply decreased as hydroxyproline contents accumulated (Figures S2C, S2D, and S2F–S2H). All patients’ synovial tissue was examined with H&E staining and IHC by two independent pathologists for further corroboration. H&E staining of the AF group showed high-grade fibrosized synovial tissue while IHC exhibited highly expression of a-SMA and Col-1 (Figure S2E). These pathological results serve as an important complement for a more precise AF diagnosis criterion.

### Cellular contribution analysis of arthrofibrosis synovium

**Figure 1:**
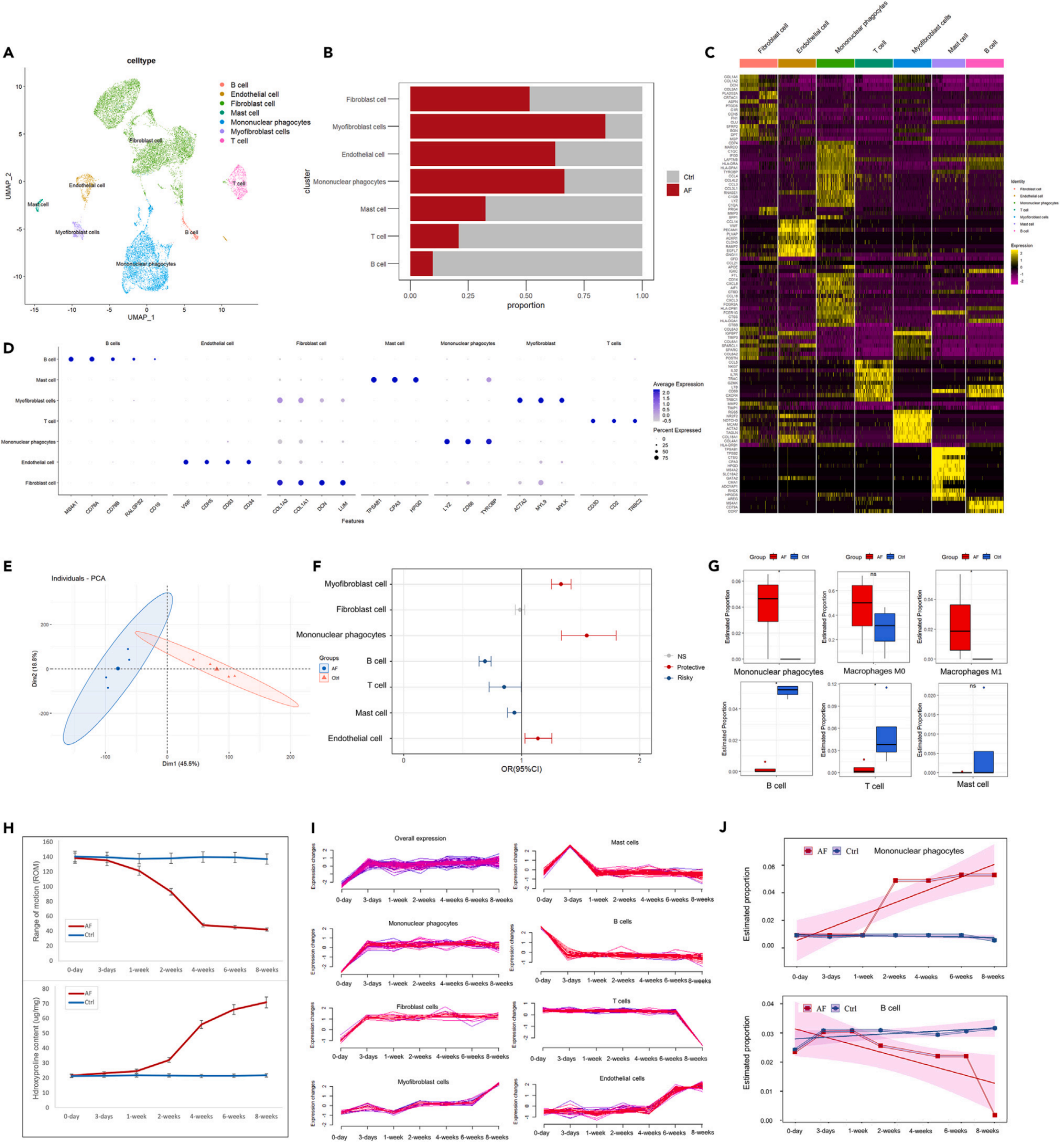
Cellular contribution analysis of arthrofibrosis synovium (A) Unsupervised clustering of single-cell RNA sequencing, visualized by uniform manifold approximation and projection (UMAP) showing major clusters. Each point in the figure represents a single cell. (B) Cell proportion comparison between the arthrofibrosis group and the non-arthrofibrosis group. (C) Heatmap of the top ten markers of each cell type. (D) Dot plot of representative marker expression in each cell type. (E) Dimension reduction of bulk sequencing data by PCA. (F) Deconvolution analysis of cell type proportions in human bulk RNA sequencing data. The OR was calculated by comparison between the arthrofibrosis group and the non-arthrofibrosis group. (G) CIBERSORT analysis of immune cell proportions in human bulk RNA sequencing data. The stars indicate a statistically significant difference, and ns represents a nonsignificant difference. (H) Range of motion (ROM) and hydroxyproline contents change over time in animal models. (I) Identified marker gene expression changes over time in the different cell types of animal models. (J) CIBERSORT analysis of immune cell proportion changes in animal models.

Based on the conformation of the pathological analysis, four of the typical synovial tissue (2 AF and 2 non-AF) were subjected to single-cell sequencing. After quality control, normalization, integration, dimension reduction, and unsupervised clustering, the scRNA-seq data were manually annotated to six different cell types including fibroblast cells, myofibroblast cells, endothelial cells, mononuclear phagocytes, mast cells, T cells, and B cells (Figure 1A; Figure S3A). The cell proportions of myofibroblast cells, endothelial cells, and mononuclear phagocytes sharply increased in the AF group, while mast cells, T cells, and B cells decreased (Figure 1B; Table 1). The heatmap and dot plot show the representative marker expression of each cell type which was used for subsequent deconvolution analysis (Figures 1C and 1D). The feature plot of the marker genes clearly shows the distribution of each cell type (Figure S3B).

**Table 1:** Overall cell count and proportion in each cell type and defined subcluster

Cell type	Subcluster	Non-AF		AF		Comparison
		Count	Cell Proportion	Count	Cell Proportion	p value
Myofibroblast cells	–	133	0.010	646	0.047	0.003
Fibroblast cells	DKK3-SLF	333	0.026	5441	0.399	7.62E-05
	CD34-SLF	1663	0.134	1563	0.115	6.05E-05
	CD55-LLF	5218	0.420	61	0.004	0.002
	Total	7214	0.581	7065	0.518	0.197
Mononuclear phagocytes	M1-like F-Mφ	8	0.001	818	0.060	1.92E-06
	M2-like F-Mφ	125	0.010	201	0.014	0.005
	I-Mφ	1197	0.096	1448	0.106	0.427
	IFN-Mφ	36	0.003	29	0.002	0.001
	IR-Mφ	200	0.016	105	0.007	0.001
	S100A8/9-Mφ	229	0.018	197	0.014	0.006
	T-Mφ	635	0.051	1544	0.113	0.005
	cDC1	91	0.007	209	0.015	0.003
	cDC2	78	0.006	172	0.014	0.008
	Total	2599	0.209	4723	0.346	0.001
T cells	Naive CD4^+^ T cells	176	0.014	23	0.001	1.88E-06
	Natural killer T cells	49	0.004	61	0.004	0.098
	T follicular helper	159	0.013	14	0.001	0.000
	CD4^+^ regulatory T cells	105	0.008	16	0.001	0.045
	CD4^+^ T helper cells 1	143	0.012	28	0.002	0.260
	CD4^+^ T helper cells 17	43	0.003	22	0.001	0.232
	CD4^+^ T helper cells 2	82	0.007	30	0.002	0.001
	Naive CD8^+^ T cells	333	0.027	83	0.006	0.018
	CD8^+^ effector T cells	155	0.012	29	0.002	0.001
	Total	1245	0.100	306	0.022	0.054
B cell	–	503	0.041	52	0.003	0.002
Mast cell	–	231	0.019	105	0.007	0.004
Endothelial cell	–	456	0.037	711	0.052	0.005
Total		12405	1	13621	1	–

To verify the cell proportion changes revealed by sc-RNA-seq, we then performed a deconvolution analysis of cell type proportions in human bulk-seq data. The principal component analysis (PCA) dimension reduction could clearly distinguish AF patients from control group patients (Figure 1E). The results of bulk-seq deconvolution analysis were consistent with scRNA-seq (Figure 1F). CIBERSORT analysis also confirmed that mononuclear phagocytes, especially M1 macrophages, were significantly increased in the AF groups. B cells and T cells were decreased in this test.

To gain further insight into the cell contribution to the pathogenesis of AF, we established an animal model. ROM and hydroxyproline contents were checked and documented at different endpoints including contralateral knee as the control 3 days after fixation, 1 week after fixation, 2 weeks after fixation, 4 weeks after fixation, 6 weeks after fixation, and 8 weeks after fixation (Figure 1H). We have also performed bulk-seq for these samples in each group. From week 2 to week 4 is the key stage of AF development which exhibits fasted hydroxyproline content accumulation and ROM loss. However, the expression of the identified marker gene was changed earlier after fixation (Figure 1I). Mononuclear phagocytes contributed the most to this pathological process which was sharply increased after 1-week of fixation, as revealed by CIBERSORT analysis (Figure 1L). B cells and T cells were primarily decreased after 6 weeks of fixation.

### Cell functional state analysis

To explore the cell functional state of AF group cells, we performed a single-cell rank-based gene set enrichment analysis of scRNA-seq data. Compared with the housekeeping gene set, the four biological functional gene sets including inflammation, cell adhesion, reactive oxygen species (ROS), and hypoxia were activated to different degrees (Figures S4A–S4C). We then calculated the average gene expression of these five genes sets in human bulk sequencing data, which verified that these four biological functions were associated with AF (Figure S4D). Animal model bulk-seq data also revealed that these biological functions were activated at the very beginning of fixation (Figure S4E).

### Cell communication and pathway analysis

**Figure 2:**
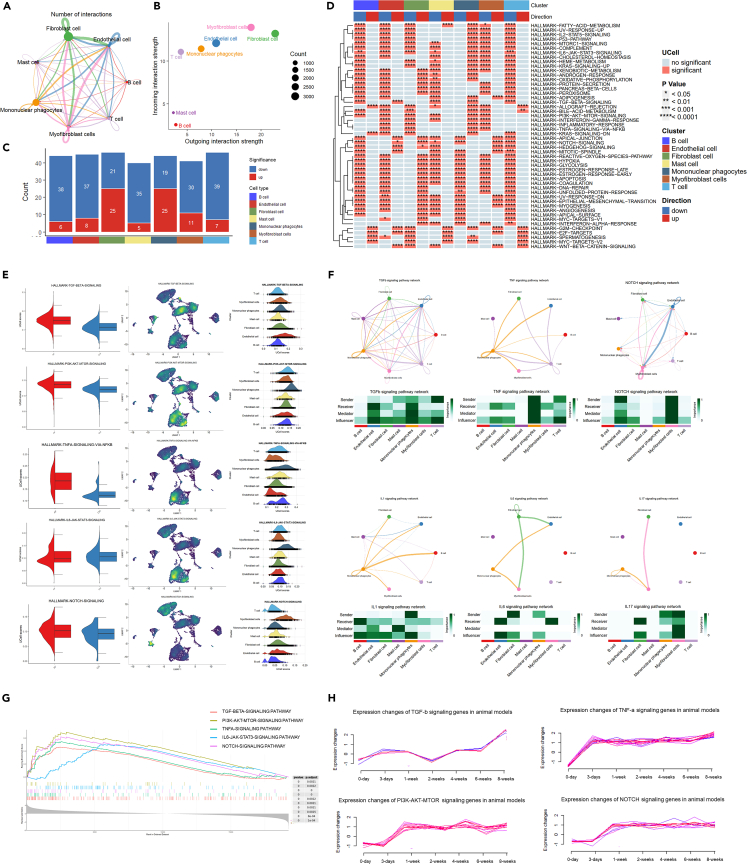
Cell communication and functional analysis (A) Circle plot of the interaction of each cell type. (B) Cell communication network construction of each cell type. (C) Numbers of significantly changing pathways in different cell types compared with the control group. (D) Changing pathways heatmap of different cell types compared with the control group. (E) Semi-violin plot, density scatterplot, and ridge plot of five key fibrotic signaling pathways including TGF-β, PI3K-Akt, TNF-α, IL6-JAK-STAT3, and NOTCH signaling pathways. (F) Circle plot and heatmap of the key fibrotic pathway network including TGF-β, TNF-α, NOTCH, IL1, IL6, and IL17 signaling pathways. (G) Gene enrichment analysis of the five key fibrotic signaling pathways. (H) Fold-line plot of the gene expression levels of four fibrotic signaling pathways.

Subsequently, we performed cell communication network construction with CellChat (Figures 2A and 2B). Fibroblasts are the most important secretory cells in the pathogenesis of AF while myofibroblasts are the major target cells. Mononuclear phagocytes, endothelial cells, and T cells are also important secretory cells and target cells. The fully fibrosis-related cell interactions are depicted by a heatmap (Figures S5A–S5C).

Pathway analysis of different cell types was then performed to uncover the heterogeneity of the synovial microenvironment in arthrofibrosis pathogenesis. The significantly changed pathways are listed in the fraction bar plot (Figure 2C) and heatmap (Figure 2D). Fibroblast cells and mononuclear phagocytes were the most important driving cells in AF development with the most upregulated pathways compared with the control group. Among them, five signaling pathways were the key fibrotic pathways reported by previous studies including TGF-β, PI3K-Akt, TNF-α, IL6-JAK-STAT3, and NOTCH (Figure 2E).^2^^,^^5^^,^^6^^,^^9^ The cell communication of these key pathways was examined and is depicted in Figure 2F. Gene enrichment analysis of bulk sequencing of humans and animals also confirmed that these pathways were all upregulated (Figures 2G and 2H). The TGF-β signaling pathway is activated in almost all cell types, and is primarily secreted by T cells and targeted to mononuclear phagocytes and endothelial cells. TNF-α signaling pathway was mainly activated in mononuclear phagocytes which also influenced T cells. The NOTCH signaling pathway was majorly activated in the myofibroblast cells and influenced itself. Other pathways were primarily activated in mononuclear phagocytes and influenced fibroblast cells and myofibroblast cells. These results may suggest that mononuclear phagocytes and T cells are driving cells of fibrosis.

### Common differential gene identification

A total of 1884 differential expressed genes (DEGs) were identified in human bulk-seq data with |logFC|>2 and adjust p value<0.05 (Figure S6A; Table S3). GO analysis of these genes revealed that their functions are related to cell adhesion, extracellular structure organization, and extracellular matrix organization. The most correlated pathway involves focal adhesion, notch signaling pathway, cell adhesion molecules cams, etc. 84 DEGs of scRNA-seq data were identified with the |logFC|>2 and adjust-pvalue<0.05 (Figure S6B; Table S4). Based on intersection analysis, 16 common DEG genes between bulk-seq and sc-RNA seq were identified. Fibrotic-related genes including ACTN1, COL6A1, MFGE8, IGFBP7, EN1, SPARC, MAP1B, EDIL3, TPM1, COL6A2, and BGN were highly expressed in fibroblast cells and myofibroblasts in the AF group (Figure S6C). Immuno-related genes including CCL3, CCL2, and CD14 were highly expressed in mononuclear phagocytes (Figure S6C). Moreover, we performed immunohistochemical scoring of cellular biomarkers in the patient cohort to verified the changes in different cell types in the AF groups (Figure S6D). We observed a significant increase in the expression of myofibroblast marker (ACAT2) and M1 macrophage markers (CD161) in the AF group compared to the control group.

### Regulatory network analysis

The regulatory network was constructed and significantly changing transcription factors between the AF group and control group were identified (Figures S7A and S7B). EN1, NR2F6, JDP2, HOXB2, and ARID3A were the top five transcription regulons in the AF group (Figures S7C–S7E).

**Figure 3:**
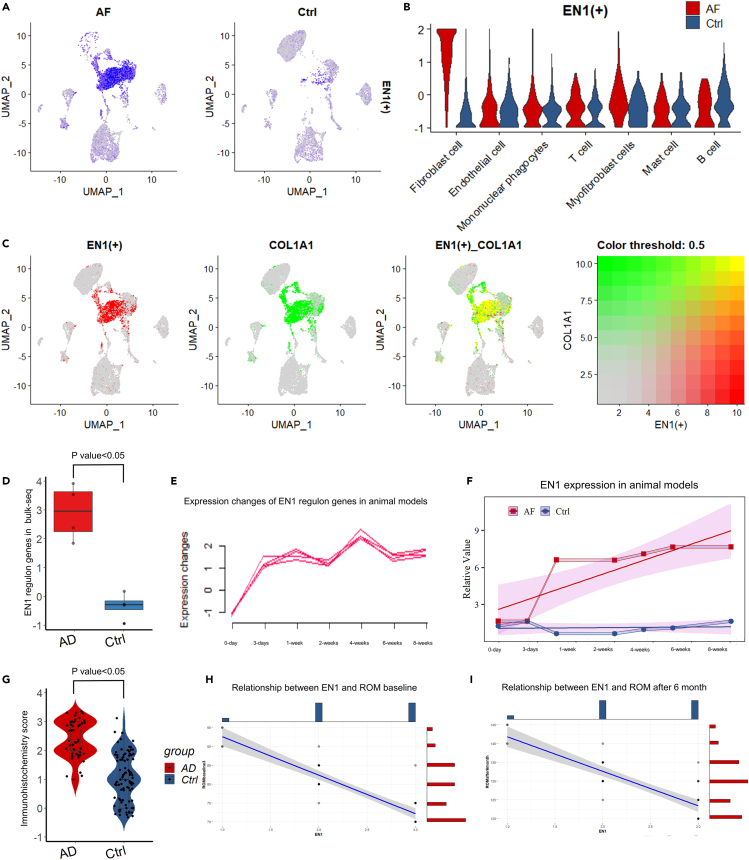
EN1 regulon analysis (A) Feature plot of EN1 regulon splitting by AF group and Non-AF group. (B) Violin plot of the EN1 regulon in different cell types. (C) EN1, COL1A1, and merged feature plots of single-cell sequencing data. (D) Boxplot of EN1 expression in human bulk sequencing data. (E) Fold line plot of EN1 regulon expression genes changing over time in the animal model. (F) Fold line plot of EN1 expression genes changing over time in the animal model. (G) Violin plot of IHC score evaluation of EN1 in clinical patients. (H) Correlation between EN1 and ROM baseline. (I) Correlation between EN1 and ROM after 6 months.

Among them, the Engrailed-1 (EN1) regulon was reported to be one of the most important transcription regulons related to fibrosis development.^17^^,^^18^^,^^19^ In our study, the EN1 regulon was significantly activated in the AF group compared with the control group (Figure 3A). It was majorly expressed in fibroblast cells (Figure 3B). The merged feature plot revealed that EN1 had a significantly greater overlap with COL1A1 (Figure 3B). Bulk sequencing data from human and animal models also confirmed that the EN1 regulon was highly expressed during AF development (Figures 3D–3F).

Moreover, we carried out further testing on 141 patients (47 AF patients group and 94 non-AF control group). Immunohistochemistry staining scores showed a significant increase in the AF groups compared with the control group (Figure 3G). The correlation analysis revealed that EN1 was negatively correlated with ROM at baseline before revision surgery (R = −0.89, p < 0.01) and 6 months after surgery (R = −0.84, p < 0.01). These results indicated that the EN1 may be the core transcription driving AF pathogenesis which is related to disease severity and prognosis.

### Fibroblast subclusters analysis

**Figure 4:**
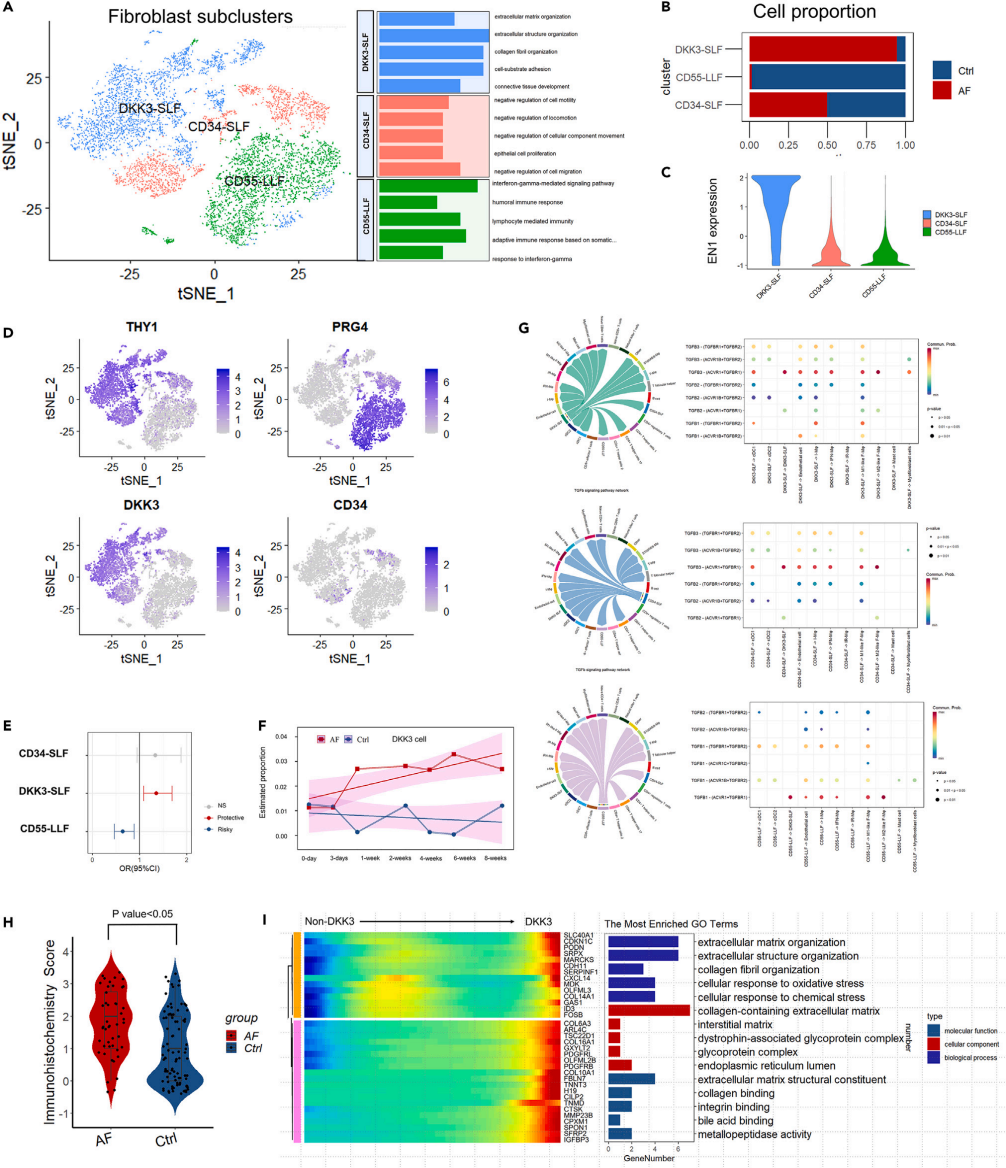
Fibroblast subcluster analysis (A) Dimensional reduction and functional enrichment of fibroblast subcluster cells. (B) Cell proportion of different fibroblast subcluster cells compared between the arthrofibrosis group and the non-arthrofibrosis group. (C) EN1 regulon expression in different fibroblast subcluster cells. (D) Feature plot of fibroblast subcluster cells. (E) Deconvolution analysis of cell type proportions in human bulk RNA sequencing data. (F) Deconvolution analysis of DKK-SLF cell proportion changes in animal models. (G) Chord diagram and dot plot of communication between different fibroblast subclusters. (H) Violin plot of IHC score evaluation of DKK3 in clinical patients. (I) Heatmap and functional enrichment analysis of significant genes that covaried across pseudotime splits in DKK3 and non-DKK3 cells.

To uncover the heterogeneity of fibroblast cells in AF, we performed data integration and dimensional reduction once again for fibroblast cells. According to previous studies,^20^^,^^21^^,^^22^^,^^23^^,^^24^ the subcluster of fibroblast cells can be manually annotated into three major groups including DKK positive sublining fibroblasts (DKK3-SLF), CD55^+^ lining fibroblasts (CD55-LLF), and CD34^+^ sublining fibroblasts (CD34-SLF) (Figure 4A). DKK3-SLF was the pathogenic fibroblast cell located in the synovial membrane’s sub-lining layer (SL) with high expression of CD34, POSTN, THY1, and EN1 (Figures 4C and 4D) in agreement with previous reports.^24^^,^^25^^,^^26^^,^^27^ Functional analysis of DKK3-SLF cell markers revealed that this cell type was correlated with extracellular matrix organization, extracellular structure organization, collagen fibril organization, cell-substrate adhesion, and connective tissue development (Figures 4A; Figure S8D). The proportion of DKK3-SLF cells was overwhelmingly increased in the AF group (Figure 4B). Deconvolution analysis of both human and animal model bulk sequencing data also confirmed the surge in DKK3-SLF cells numbers (Figures 4E and 4F). DKK3-SLF started to rapid proliferation at approximately one-week time points. Immunohistochemistry staining scores of DKK3 showed a significant increase in the AF groups compared with the control group (Figure 4H).Single-sample gene set enrichment analysis (ssGSEA) also revealed that DKK3-SLF exhibited the most significantly up-regulated fibrotic pathway (Figure S8A). As the key pathway, the TGF-β signaling pathway was increased in the DKK3-SLF compared with the other fibroblasts (Figures S8B and S8C). Cell-cell communication analysis showed that DKK3-SLF had the strongest secretory function of TGF-β3 while other fibroblasts primarily secreted TGF-β1 (Figure 4G). TGFBR1 was the most important receptor across all kinds of fibroblasts, which was consistent with our previous studies.^28^ Functional enrichment analysis of significant genes that covaried across pseudotime showed a high correlation with AF development (Figure 4J). These results suggested that the DKK3-SLF was the key cell type in AF pathogenesis.

### Myofibroblast analysis

**Figure 5:**
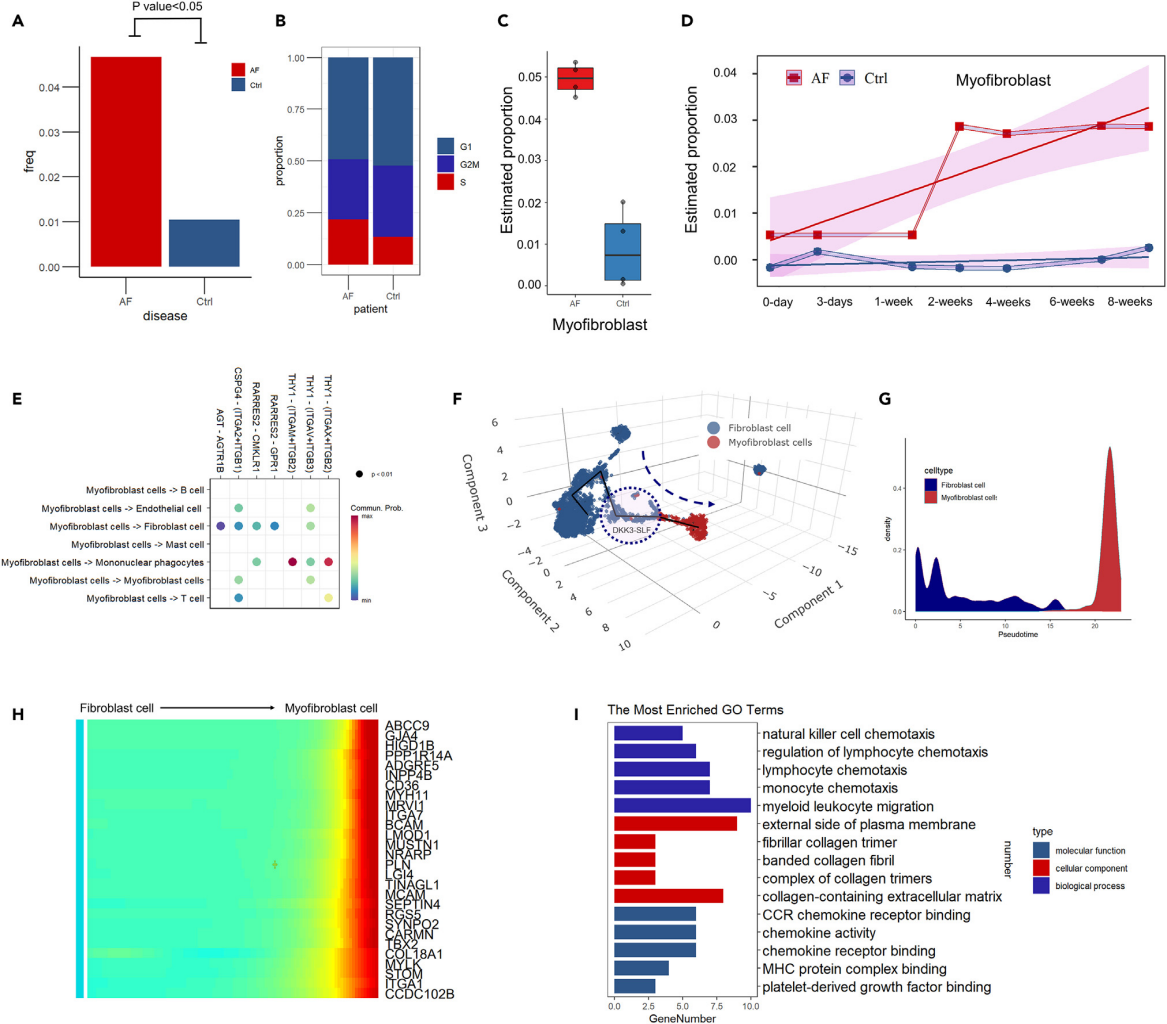
Myofibroblast subcluster analysis (A) Comparison of the myofibroblast cell proportion between the AF and control groups. (B) Fraction of total cells present in each cell stage. (C) Estimation of Myofibroblast proportions in human bulk RNA sequencing data. (D) Estimation of Myofibroblast proportions changing over time in the animal model. (E) Fibrotic-related pathway network analysis of the myofibroblast subcluster. (F) 3D pseudotime trajectory analysis of myofibroblasts combined with fibroblasts. (G) Pseudotime trajectory analysis of myofibroblasts combined with fibroblasts. (H) Heatmap of significant genes that covaried across a pseudotime split in myofibroblasts and fibroblasts. (I) Functional enrichment analysis of significant genes that covaried across a pseudotime split in DKK3 and non-DKK3 cells.

Myofibroblasts are the major effector cells in AF. The number of myofibroblast cells was significantly increased in the AF group (Figure 5A). These myofibroblast cells were more active in proliferation with approximately twice the number of S-stage cells compared with the control group (Figure 5B). The surge of myofibroblast cells was also confirmed by both human and animal models with deconvolution analysis (Figures 5C and 5D). The number of cells began increasing around one week after fixation. Fibrotic-related biological processes were activated in AF including apical junction, epithelial-mesenchymal transition, hypoxia, and ROS (Figures S9A and S9B). Cell communication analysis of myofibroblasts revealed that they are important secretory cells targeted to fibroblast and mononuclear phagocytes (Figure 5E; Figure S9C). Fibrotic-related signaling pathways were activated in myofibroblasts including the AGT signaling pathway, CSPG4 signaling pathway, CHEMERIN signaling pathway, and THY1 signaling pathway.

To investigate the relationship between myofibroblasts and fibroblasts, we then performed 3D and 2D pseudotime trajectory analysis (Figures 5F and 5G). A clear trajectory from fibroblasts to myofibroblasts was shown with the DKK3-SLF in the transitional zone. This finding could indicate that the DKK3-SLF was the major fibroblast subcluster which differentiated to the myofibroblast. Significant genes covaried across a pseudotime split in myofibroblasts and fibroblasts cells, as shown in Figure 5H. Functional enrichment analysis indicated that these genes were correlated with monocyte chemotaxis and collagen-containing extracellular matrix.

### Mononuclear phagocyte subclusters analysis

**Figure 6:**
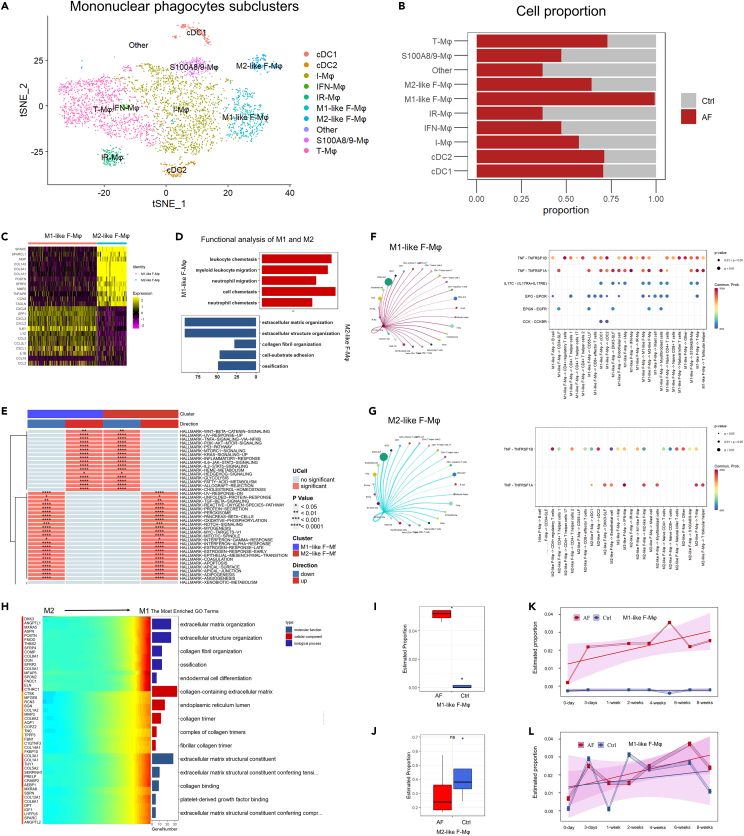
Mononuclear phagocyte subcluster analysis (A) Dimensional reduction of mononuclear phagocytes. (B) Cell proportion of different fibroblast subcluster cells compared between the arthrofibrosis group and non-arthrofibrosis group. (C) Heatmap of marker genes of M1-like F-Mφ and M2-like F-Mφ. (D) Functional enrichment analysis of marker genes of M1-like F-Mφ and M2-like F-Mφ. (E) Heatmap of significantly changed pathways of myofibroblasts compared with the control group. (F) Cell communication analysis of M1-like F-Mφ. (G) Cell communication analysis of M2-like F-Mφ. (H) Heatmap and functional enrichment analysis of significant genes that covaried across a pseudotime split in M1-like F-Mφ and M2-like F-Mφ. (I and J) Estimation of M1-like F-Mφ and M2-like F-Mφ proportions in human bulk RNA sequencing data. (K and L) Estimation of M1-like F-Mφ and M2-like F-Mφ proportions changing over time in the animal model.

Based on previous studies,^24^^,^^29^^,^^30^^,^^31^ the mononuclear phagocytes were annotated to 10 different subclusters including conventional type 1 dendritic cells (cDC1), conventional type 2 dendritic cells (cDC2), M1-like fibrotic macrophage (M1-like F-Mφ), M2-like fibrotic macrophage (M2-like F-Mφ), interferon-stimulated macrophages (IFN-Mφ), S100A8/9 high macrophages (S100A8/-Mφ), inflammatory macrophages (I-Mφ), immune regulated macrophages (IR-Mφ), transitional macrophages (T-Mφ) and others (Figure 6A). M1-like F-Mφ highly expressed canonical M1 macrophage markers and M2-like F-Mφ highly expressed canonical M2 macrophage markers (Figure 6C). Functional enrichment analysis revealed that the M1-like F-Mφ were correlated with cell chemotaxis while the M1-like F-Mφ were correlated with extracellular matrix organization, collagen fibril organization, and cell-substrate adhesion (Figure 6C). The M1-like F-Mφ number was overwhelmingly increased in the AF synovium (Figure 6B). Deconvolution analysis of both human and animal model bulk sequencing data also confirmed the surge in the M1-like F-Mφ number (Figures 6I and 6K). The M1-like F-Mφ started proliferating just after fixation surgery. However, the M2-like F-Mφ were not statically significant in this test (Figures 6J and 6L).

Single-sample gene set enrichment analysis (ssGSEA) also revealed that the M1-like F-Mφ were highly associated with many fibrotic-related biological processes and signaling pathways (Figure 6E). Cell communication analysis depicted the difference in cell interaction network between M1-like F-Mφ and M2-like F-Mφ (Figures 6F and 6G). M1-like F-Mφ exhibited a higher capacity for secreting TNF-α, IL17, EPO, EPGN, and CCK. These cytokines may contribute to AF development. Pseudotime trajectory analysis also showed that the significant genes that covaried across a pseudotime split in M1-like F-Mφ and M2-like F-Mφ were related to many fibrotic biological processes.

### T cell subcluster analysis

**Figure 7:**
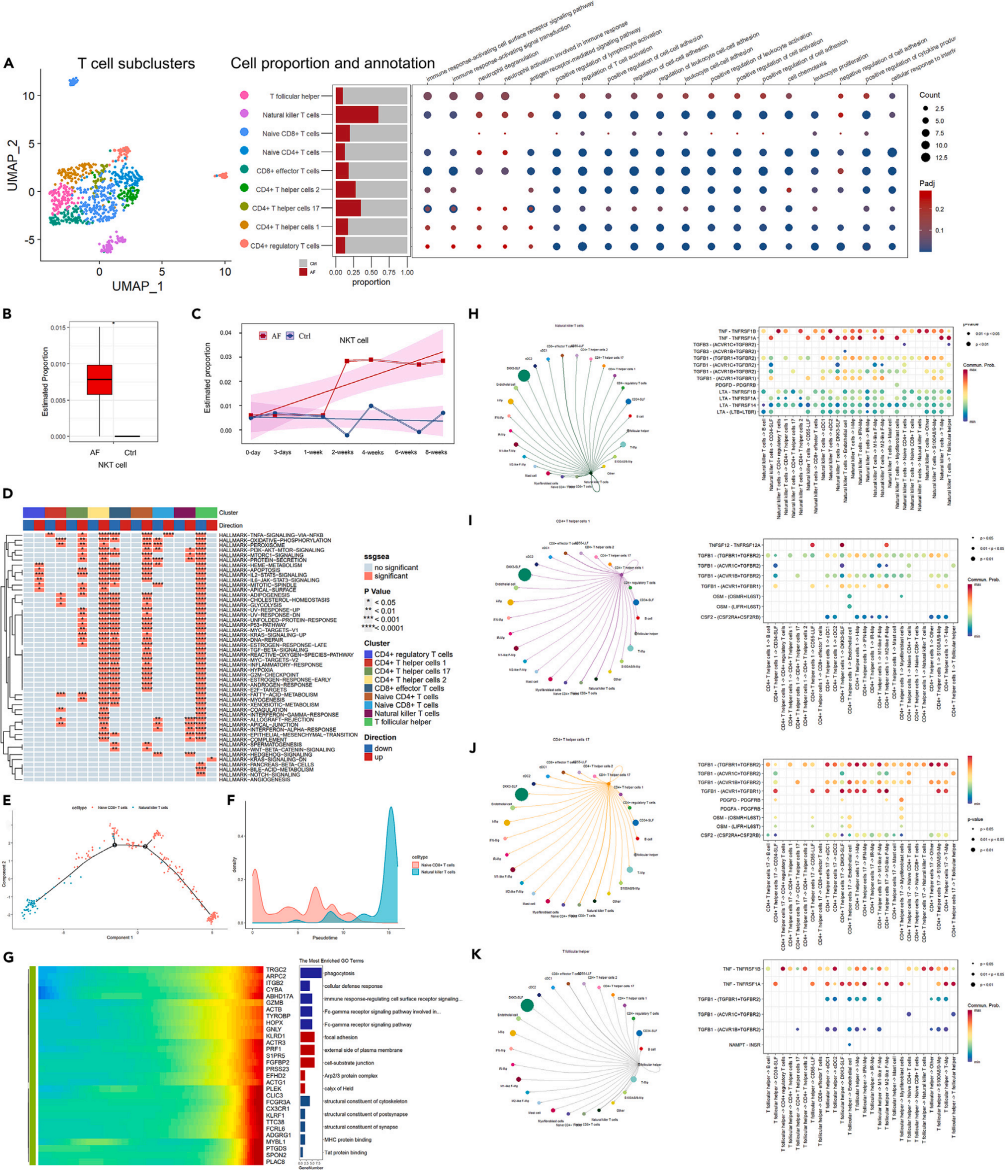
T cell subcluster analysis (A) Dimensional reduction, cell proportion, and functional annotation dot plot of T cell subclusters. (B) Estimation of NKT-cell proportions in human bulk RNA sequencing data. (C) Estimation of NKT-cell proportions changing over time in the animal model. (D) Heatmap of significantly changed pathways of different T cell subclusters compared with the control group. (E and F) Pseudotime trajectory analysis of NKT and naive CD8^+^ T cells. (G) Heatmap and functional enrichment analysis of significant genes that covaried across a pseudotime split in NKT and naive CD8^+^ T cells. (H–K) Cell communication analysis of fibrotic-related T cell subclusters, including NKT, Th1, Th17, and Tfh cells.

Based on previous studies,^23^^,^^31^^,^^32^ the T cells were annotated to 9 different subclusters including T follicular helper (Tfh), natural killer T cells (NKT), naive CD8^+^ T cells, naive CD4+T cells, CD8^+^ effector T cells, CD4^+^ T helper cells 1 (Th1), CD4^+^ T helper cells 2 (Th2), CD4^+^ T helper cells 17(Th17), and CD4^+^ regulatory T cells (Treg) (Figure 7A). Among them, NKT is the only cell type increasing in the AF group. Deconvolution analysis of both human and animal model bulk sequencing data also confirmed the increase in NKT cell numbers (Figures 7B and 7C). The NKT cell number began to increase at approximately the two-week time point (Figure 7C). Single-sample gene set enrichment analysis (ssGSEA) revealed that NKT cells exhibited a high association with many fibrotic-related biological processes and signaling pathways (Figure 7D).

We also performed the pseudotime trajectory analysis on naive CD8^+^ T cells to identify significant genes (Figures 7E–7G). Functional enrichment analysis revealed that the significant genes of NKT cells were correlated with a structural constituent of the cytoskeleton, focal adhesion, and immune response-regulating cell surface receptor signaling pathways (Figure 7G).

Cell communication analysis constructed different cell interaction networks of fibrotic-related T cell subclusters including NKT, Th1, Th17, and Tfh cells (Figures 7H–7K). These cells manifested a different pattern of cell interactions.

## Discussion

In this study, we developed an integration pipeline using deconvolution analysis of human, animal modal, and public data. Compared with previous studies using only clinical criteria, we combined both clinical and pathological criteria in this study for a more precise AF diagnosis.

Based on the integrated analysis, the microenvironment of the AF joint is shown here which was orchestrated by fibroblast cells, myofibroblast cells, endothelial cells, mononuclear phagocytes, mast cells, T cells, and B cells. A series of fibrotic functional mediators of inflammation, cell adhesion, ROS, and hypoxia were explicitly activated in this microenvironment which drives AF pathogenesis. The TGF-β, TNF-α, PI3K-AKT, and NOTCH pathways were also upregulated in this process. According to previous studies, TGF-β and TNF-α produced by fibroblast and mononuclear phagocytes can promote ROS and suppress antioxidant enzymes.^33^^,^^34^^,^^35^ A high level of ROS production stimulates TGF-β and TNF-α secretion which forms a positive feedback cycle.^36^ It also upregulates and stabilizes the hypoxia-inducible factor-1 (HIF) which leads to hypoxia in synovial tissue.^37^ These two biological processes will cause cell death and tissue damage which lead to an increase in inflammatory cytokines and collagen deposition.^36^^,^^37^^,^^38^ Moreover, inflammation and cell adhesion in the tissue reduce vascularity, which results in permanent hypoxia and another positive feedback loop of AF.^38^^,^^39^ These four biological processes are closely related and vigorously promoted in AF development.^2^

In addition, we identified and verified EN1 as a key transcription factor of AF that was correlated with disease severity and clinical prognosis. Recent studies revealed that EN1 is a key amplifier of the TGFβ signaling pathway, which contributes to fibrotic effects.^17^^,^^18^^,^^19^ The regulon of EN1 was activated by mechanical tension and drove cytoskeleton organization. Preventing EN1 activation may yield wound regeneration without fibrosis formation. Thus, EN1 could be a reliable candidate for the clinical diagnosis, prognosis, and treatment of AF.

Notably, we figured out DKK3-SLF as a special pathogenic fibroblast cell subcluster in AF development. It was the major fibroblast cell type identified in AF. Based on recent studies, DKK3-SLF cells are pathogenic and inflammation-associated fibroblast subclusters that exist across various tissues and lead to different fibrosis diseases.^21^^,^^24^^,^^25^^,^^26^^,^^27^ This cell type was located in the synovial membrane’s sublining layer (SL), which drove inflammation and ECM remodeling by producing cytokines (TGF-β1, TGF-β3, IL1, etc.). We have also proven that DKK3-SLF cells are Engrailed-1 lineage–positive fibroblasts (EPFs), which are generally believed to predominate in ECM synthesis and organization.^40^ Compared with lining layer fibroblast cells (CD55-LL, THY+) and other SLF cells, DKK3-SLF cells were proven to be nondestructive to the bone and cartilage. This may explain why the bones are intact even in severe AF patients, which is contrary to rheumatoid arthritis. In addition, a higher proportion of myofibroblast cells was also represented in the AF, which was consistent with previous studies.^1^^,^^41^ Our study confirmed that myofibroblasts are derived from fibroblast in the synovial microenvironment, especially DKK3-SLF. Mechanical forces may drive these fibroblasts to differentiate into myofibroblasts by activating the EN1 regulon.^42^

Recent researchers have illustrated that mononuclear phagocytes, especially activated M1 macrophages, are important in AF initiation and maintenance.^43^^,^^44^ Clinical studies have demonstrated a positive correlation between M1 macrophage tissue density and preoperative patient-reported KSS outcomes.^44^ In this study, we identified the M1-like F-Mφ subcluster as the key driver cell type of AF pathogenesis. It is widely accepted that macrophages are not only permanent cells, that can transfer from M0 to M1/M2. The imbalance of M1/M2 macrophages accounts for a low-grade inflammatory state and fibrotic changes in the knee joint.^30^ We have elucidated that the number of M1-like F-Mφ is significantly increased and activated at the very beginning of fixation. The significant polarization of M1 results in the promotion of inflammatory chemokines and cytokines, including TGF-β, TNF-α, IL-1, and IL-6, which lead to inflammation and collagen accumulation in the AF synovial microenvironment. It is worth noting that previous studies have indicated a positive correlation between M1 cell density and BMI,^45^ which may be attributed to adipose-related inflammation impacting macrophage populations.

In contrast to previous studies, we did not observe elevated levels of mast cells, B cells, or T cells in patients with AF in our study. This may be attributed to our relatively small sample size, and further validation is necessary to clarify this conclusion. However, we did identify an increase in the proportion of NKT cells. To date, there is little research into the relationship between NKT cells and AF development. Our study provided evidence that NKT cells play an important role in the AF synovial microenvironment, which fills this blank. Studies on liver fibrosis have shown that NKT cells serve as central components of fibrosis and the inflammatory response.^32^^,^^46^^,^^47^ It was reported that NKT cells were significantly increase in fibrosis and plastically modulated the immune response.^48^ Further study of NKT cells in AF is warranted which could open new avenues for reliable therapeutic target identification and AF treatment.

Overall, our study characterizes the components and heterogeneity of AF. We also identified EN1, which could be a reliable therapeutic target and clinical diagnostic biomarker for AF. These results contribute to the understanding of AF and other fibrotic diseases, which may open new avenues for therapeutic target identification and AF treatment.

### Limitations of the study

This study has certain limitations. First, we attempted to ensure consistency in tissue sampling by requesting surgeons to obtain samples from the precise location in the joint, thereby minimizing potential bias arising from sampling different tissue types. However, for revision patients, the anatomical structures within the joint are more complex, and obtaining perfect samples may be challenging, which may have introduced some bias. Second, compared with previous studies,^49^ we have observed more pronounced differences in histological and IHC staining between the fibrosis patients’ synovial tissues and the control group in the single-cell sequencing approach we employed. This may introduce potential selection bias in the single-cell sequencing data. We will increase the sample size for further validation of the AF in the future work.

## STAR★Methods

### Key resources table

**Table:** 

REAGENT or RESOURCE	SOURCE	IDENTIFIER
**Antibodies**		

α-SMA antibody	Abcam	RRID: AB_32575
Col1 antibody	Abcam	RRID: AB_34710
DKK3 antibody	Abcam	RRID: AB_187532
CD117 antibody	Abcam	RRID: AB_32363
CD3 antibody	Abcam	RRID: AB_135372
CD161 antibody	Abcam	RRID: AB_234107
CD20 antibody	Abcam	RRID: AB_9475
EN1 antibody	Abcam	RRID: AB_108598

**Deposited data**		

Arthrofibrosis single cell sequence data	This study	CNP0004530

**Software and algorithms**		

R software (Version 4.2.0)	R core team	https://www.r-project.org/
R package limma (Version 3.46.0)	Ritchie^50^	R-project.org/paokago-limma
MuSiC (Version 0.2.0)	Wang^15^	https://github.com/xuranw/MuSiC
CIBERSORT (Version 2.18.0)	Newman^16^	https://github.com/jason-weirather/CIBERSORT
monocle (Version 1.4.0)	Qiu^51^	https://github.com/cole-trapnell-lab/monocle-release
CellChat (Version 1.6.0)	Jin^52^	https://github.com/sqjin/CellChat
irGSEA (Version 1.1.3)	Chuiqin	https://github.com/chuiqin/irGSEA/
Ucell (Version 1.3)	Andreatta^53^	https://github.com/carmonalab/UCell
singscore (Version 1.2.2)	Bhuva^54^	https://github.com/DavisLaboratory/singscore
AUCell (Version 1.12.0)	Aibar^55^	https://github.com/aertslab/AUCell
GSVA (Version 1.38.2)	Hänzelmann^56^	https://github.com/rcastelo/GSVA
Mfuzz (version 2.50.0)	Kumar^57^	https://github.com/a-velt/Mfuzz_RNAseq
ggstatsplot (version 0.7.1)	Patil^58^	https://github.com/IndrajeetPatil/ggstatsplot
SCENIC (Version 1.3.1)	Van de Sande and Aibar^55^	https://github.com/scenic-views/scenic
Seurat (Version 4.3.0)	Satija Lab and Collaborators^59^	https://satijalab.org/seurat/

### Resource availability

#### Lead contact

Further information and requests for resources and reagents should be directed to and will be fulfilled by the lead contact, Yixin Zhou (orthoyixin@yahoo.com).

#### Materials availability

This study did not generate new unique reagents.

### Experimental model and participant details

#### Patients and sample collection

We have carefully inspected the records of 705 patients who underwent revision total knee arthroplasty (TKA) surgery from January 2010 to January 2021. The baseline data of each patient were collected including sex, age, body mass index (BMI), range of motion (ROM), and X-ray imaging. This study enrolled 47 patients with AF based on both inclusive and exclusive criteria (Table S1).^8^^,^^60^^,^^61^ We then matched another 94 patients with the AF group by their gender and age (Table S2). The synovial tissue of all patients was meticulously sampled by clinical experts, with efforts made to ensure sampling from the same anatomical location within the knee joint synovium. The tissue samples underwent standardized pathological examination by two independent pathologists to determine or exclude fibrosis. For single cell sequencing, we selected two patients with severe AF, characterized by a range of motion (ROM) less than 70° in flexion, and two patients with an ROM over 120° in flexion as controls. We have meticulously determined that the limited range of movement in these patients was not due to ectopic ossification or prosthesis obstruction, which may affect joint activity, and we excluded joint activity limitations caused by incorrect prosthesis positioning or size, metal hardware, ligament reconstruction, infection (septic arthritis), pain, complex regional pain syndrome (CRPS), or other specific reasons or preoperative factors which affected the ROM. At the time of surgery, posterior capsular tissues were harvested intraoperatively under sterile conditions upon visual verification of the anatomical provenance of the sample by the operating surgeons. Samples were fixed by immersion in neutral-buffered 10% formalin for 48 h at room temperature, and processed into paraffin per standard histology protocols.^44^ The workflow of this study is shown in Figure S1. This work was approved by the Ethics Committee of Jishuitan Hospital (No.201811-09) with registration.

#### Animal model establishment

The animal model was established under the guidance of the Jishuitan Hospital’s animal research ethics institution. The Jishuitan Hospital’s animal research ethics institution provided guidance for the establishment of the animal model (No.202011-02). A total of 24 New Zealand white rabbits, weighing 2.5kg + 0.5kg, were randomly divided into six groups. Following established protocols, 1.2 mm Kirschner wires (K-wires) were used to immobilize the right knees of each rabbit in the six groups, while the left knees served as control.^62^^,^^63^^,^^64^ At the endpoints of 3 days, 1 week, 2 weeks, 3 weeks, 4 weeks, and 8 weeks for these six groups, the rabbits were humanely euthanized by intravenous administration of 20% urethane (5 g/kg). After the K-wires were removed, the range of motion (ROM) of the fixed and control knees was measured as per previous studies.^65^ A consistent force of 5 N was applied to the looped wire hooked on the distal leg, which was 8 cm distal from the proximal tibia joint surface, and the angle of the femur and tibia was measured as the ROM. The synovium in the knee cavity was meticulously collected for further analysis.

### Method details

#### Hydroxyproline content determination and immunohistochemistry evaluation

We utilized another 20 mg (wet weight) of each synovial tissue sample to determine the hydroxyproline concentration. As described,^62^ the specimens were subjected to hydrolysis with 6 mol/L HCl at a temperature of 130°C for a period of 12 h, and were subsequently neutralized using 2.5-N NaOH with the aid of methyl red as an indicator. Following the addition of 1 mL of chloramine T, all of the tissue samples along with four known hydroxyproline standards were incubated for a duration of 20 min at room temperature. Afterwards, 1 mL of p-dimethylaminobenzaldehyde solution was added to both the samples and the standards. The absorbance of the solution was determined at a wavelength of 558 nm using a spectrophotometer. Utilizing the standard curve, the hydroxyproline content of the sample was calculated.

The remaining synovial tissue was preserved in a 10% buffered formalin solution before being paraffin-embedded. Hematoxylin/eosin (HE) staining was used to assess the severity of AF in slices that were 4 μm thick. Immunohistochemical stains were carried out utilizing an automated immunostainer (Autostainer 720, Labvision) in accordance with standard heat-induced epitope retrieval and the avidin-biotin-peroxidase complex method. The primary antibodies used in this study includedα-SMA antibody (1:500,ab32575, Abcam), Col1 antibody (1:500, ab34710, Abcam), DKK3 antibody (1:500, ab187532), CD117 antibody (1:100, ab32363), CD3 antibody (1:300, ab135372), CD161 antibody (1:100, ab234107), CD20 antibody (1:100, ab9475) and anti-EN1 antibody (1:50, ab108598, Abcam). The semi-quantitative immunohistochemistry is carried out independently by two pathologists using a high-power optical microscope at 40× magnification to score the staining intensity (0 indicating negative staining, 1 indicating light yellow staining, 2 indicating light brown staining, and 3 indicating dark brown staining), with the final score being the mean value of the obtained scores. All IHC scoring has undergone standardization to enhance comparability between different groups.

#### Single-cell RNA sequencing of human synovium

Based on the previous studies,^20^ four typical synovial tissues (two AF group patients and two control group patients) were mechanically minced and enzymatically digested with Liberase TL (100 g/mL; Roche) and DNAse I (100 g/mL; Roche) for 30 min at 37°C. Red Blood Cell Lysis solution was used to lyse erythrocytes after fetal calf serum was used to stop the digestion process (Milteny Biotec). The LUNA automated cell counter was used to wash and count the cells (Logos Biosystems). Using the Chromium Single Cell 3′ GEM, Library & Gel Bead Kit v3, the Chromium Chip B Single Cell Kit (10 Genomics), and the Chromium controller (all 10 Genomics), a total of 15 000 unsorted synovial cells per patient were prepared for single cell analysis. On the Illumina NovaSeq instrument, libraries were sequenced to a depth of 20 000–70 000 reads per cell. The reads were demultiplexed, and aligned to the Ensembl reference build GRCh38.p13, and the unique molecular identifiers were collapsed using CellRanger (V.2.0.2) from 10X Genomics.

#### Single-cell data analysis

The sc-RNA seqdata were analyzed and annotated with a standard protocol.^66^ In short, the obtained expression data were loaded with the Read10X function in the R package Seurat (Version 4.0.1).^59^ Quality control was performed and low-quality cells were filtered based on the following criteria：(1) total UMI counts of no more than 1,000; (2) gene numbers no higher than 500; and (3) mitochondrial gene percentage of greater than 20%. With this, a total of 26026 cells were obtained with 13621 cells from AF group patients’ synovium samples and 12405 cells from non-AF group patients’ synovium samples. Data integration was performed with the Harmony(Version 0.1)^67^ and 2000 highly variable genes were identified after data normalization and scaling. We used the top 20 PCs for dimensional reduction and cell clustering with a resolution of 0.8. Cell annotation was accomplished manually based on the previous guidelines and the cell markers in the CellMarker 2.0 database.^66^^,^^68^

#### Public data collection and preprocessing

The gene expression chip data GSE135854 were downloaded from the NCBI Gene Expression Omnibus web resource. This public dataset involved 4 AF patients after TKA and 4 control group patients after TKA.^69^ The differential expression genes between the AF-group and the non-AF group were calculated with R package limma (Version 3.46.0)^50^ of R software (Version 4.2.0).

#### Bulk RNA sequencing of rabbit synovium

Total RNA was extracted from the 24 rabbits’ synovial tissue samples following the manufacturer’s instructions (Invitrogen) and genomic DNA was removed using Dnase I (TaKara). Then, RNA quality was assessed using an Agilent 2100 Bioanalyser and quantified using an ND-2000 (NanoDrop Technologies). RNA-seq transcriptome library was prepared in accordance with the TruSeqTM RNA sample preparation Kit from Illumina (San Diego, CA). SeqPrep and Sickle were used to trim and quality-check the raw paired-end reads. Each sample’s mapped reads were put together using a reference-based method by StringTie.

#### Deconvolution analysis of bulk RNA sequencing data

The cell type proportions in bulk RNA sequencing data were calculated with the R package MuSiC (Version 0.2.0)^15^ by deconvolution analysis. The input for the signature genes in MuSiC was generated through differential gene analysis utilizing Seurat’s one-tailed Wilcoxon rank-sum test. To account for variable expression of genes across subtypes, the top 20 signature genes were carefully selected calculated by the Seurat FindAllMarkers function. With the specific marker genes of single-cell sequencing data, the proportions of characterized cell types were estimated.

Moreover, we also utilized CIBERSORT^16^ for the immune cell analysis of human bulk seq data. CIBERSORT is a tool that utilizes the principle of linear support vector regression to perform deconvolution analysis on the expression matrices of human immune cell subtypes. This tool is applicable for both chip expression matrices and sequencing expression matrices, and its performance in deconvolution analysis of unknown mixtures and expression matrices containing similar cell types outperforms other methods such as LLSR, PERT, RLR, MMAD, and DSA. In our analysis of synovial tissue data, we selected the default LM22 gene expression signature set provided by CIBERSORT, which includes 22 immune cell subtypes.

#### Pseudotime trajectory analysis

We applied R package monocle (Version 2.18.0)^51^ to pseudotime trajectory analysis for fibroblast cells, mononuclear phagocytes, and T cells. The most significantly changing genes were identified and aggregated with comparable trends across pseudotime by the module “plot pseudotime heatmap”. The pseudotime trajectory analysis for the myofibroblasts was performed with the R package Monocle3 (Version 1.2.9).^70^ Default parameters were used to complete these tasks.

#### Cell communication analysis

Cell communication analysis of the sc-RNA seq data was performed by the R package CellChat (Version 1.4.0).^52^ The CellChatDB of 1,939 validated molecular interactions were involved in this study. In details, we extracted the raw count expression matrix from the single-cell sequencing results, where the rows represent gene names and the columns represent cell names. We then utilized the normalizeData function provided by CellChat for standardization. Next, we employed the identifyOverExpressedGenes function to identify over-expressed receptors and ligands, and projected them onto a protein-protein interaction network. For each interaction pair, CellChat assigns a probability value and calculates its significance through random computation, taking into consideration both the expression matrix and prior knowledge of interactions.

#### Functional enrichment analysis

We applied the R package ClusterProfiler (Version 4.0)^71^ for Gene ontology (GO) analysis Gene Set Enrichment Analysis (GSEA), and Kyoto Encyclopedia of Genes and Genomes (KEGG) analysis. Gene signatures were also scored by the R package Ucell (Version 1.3),^53^ singscore (Version 1.2.2),^54^ AUCell (Version 1.12.0),^55^ GSVA (Version 1.38.2),^56^ and irGSEA (Version 1.1.3).

#### Regulatory network establishment

The cellular regulatory network of AF was constructed by transcription factors (TFs) profiling using both the R package SCENIC(Version 1.3.1) by R software (Version 4.0.5) and pySCENIC (version 0.11.2) by python (Version 3.8) software.^55^ A single-cell sequencing read-count matrix was used as the input data. Subsequently, network inference analysis was accomplished in accordance with the standard SCENIC workflow (http://scenic.aertslab.org).

#### Online website database construction

The online website arthrofibrosis database (AFDB) were constructed with R package shiny (https://github.com/rstudio/shiny). The single cell data were visualized with the R package CerebroAPP (Version 1.3) (https://github.com/romanhaa/cerebroApp).^72^

### Quantification and statistical analysis

All statistical analyses were performed by R software (Version 4.0.5). Soft clustering was calculated with the R package Mfuzz (version 2.50.0).^57^ The scatter plots were depicted with the R package ggstatsplot (version 0.7.1).^58^ For all the analyses, the p-value<0.05 denoted a significant difference in this study.

## Data Availability

• The key information of single cell sequencing data and bulk sequencing are available in the arthrofibrosis database (AFDB, https://jst2023.shinyapps.io/afdb). The data that support the findings of this study have been deposited into CNGB Sequence Archive (CNSA) of China National GeneBank DataBase (CNGBdb) with accession number CNP0004530. • Code of this paper was deposited at https://github.com/chenxi199506/AFDB. • Other data support the findings of this study are available from the corresponding author upon reasonable request.

## Supplemental information

Document S1. Figures S1–S9 and Tables S1, S2, and S4

Table S3. Differential expressed genes of bulk sequencing data, related to Figure 1
