# Supplementary material for: Single-cell and bulk tissue sequencing unravels the heterogeneity of synovial microenvironment in arthrofibrosis
Source: iScience. 2023 Jul 13;26(9):107379. doi: 10.1016/j.isci.2023.107379 (PMC10495645; doi:10.1016/j.isci.2023.107379)
Supplement: Document S1. Figures S1–S9 and Tables S1, S2, and S4 [file mmc1.pdf]

iScience, Volume *26*

Supplemental information

Single-cell and bulk tissue sequencing unravels the heterogeneity of synovial microenvironment in arthrofibrosis

Xi Chen, Lihua Gong, Cheng Li, Siyuan Wang, Ziyuan Wang, Ming Chu, and Yixin Zhou

Supplementary Figure

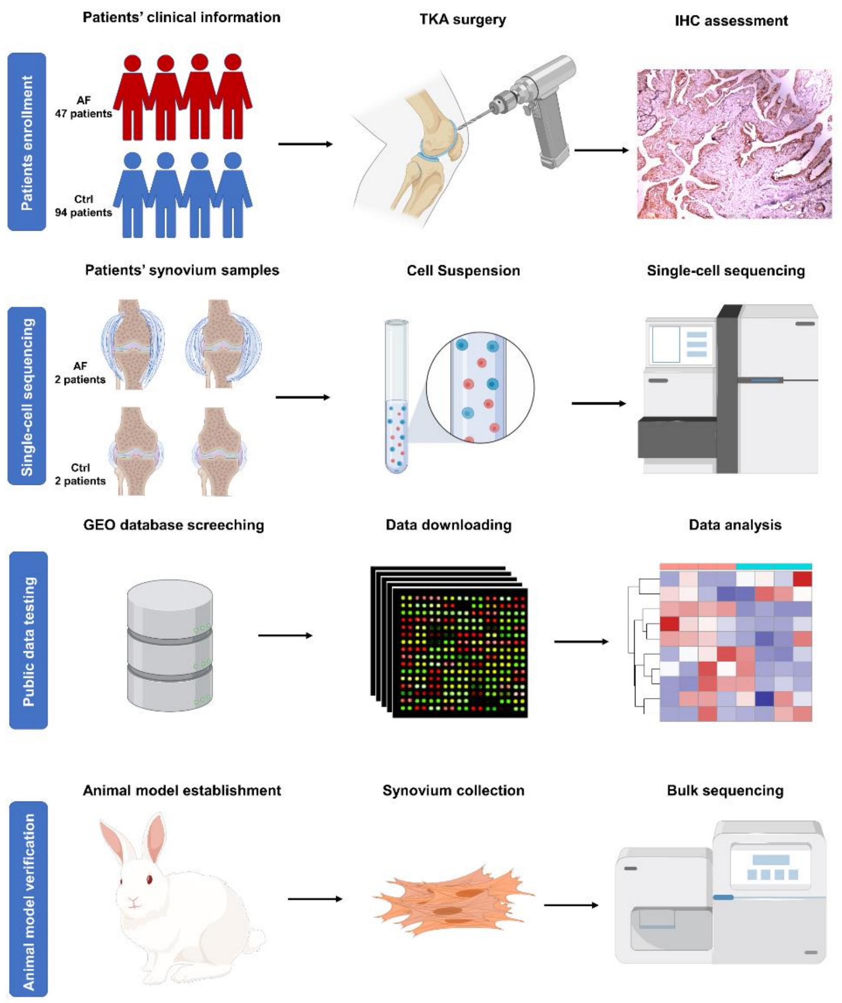

Supplementary fig. 1 Flow chart describing the overall study design, Related to Figure 1.

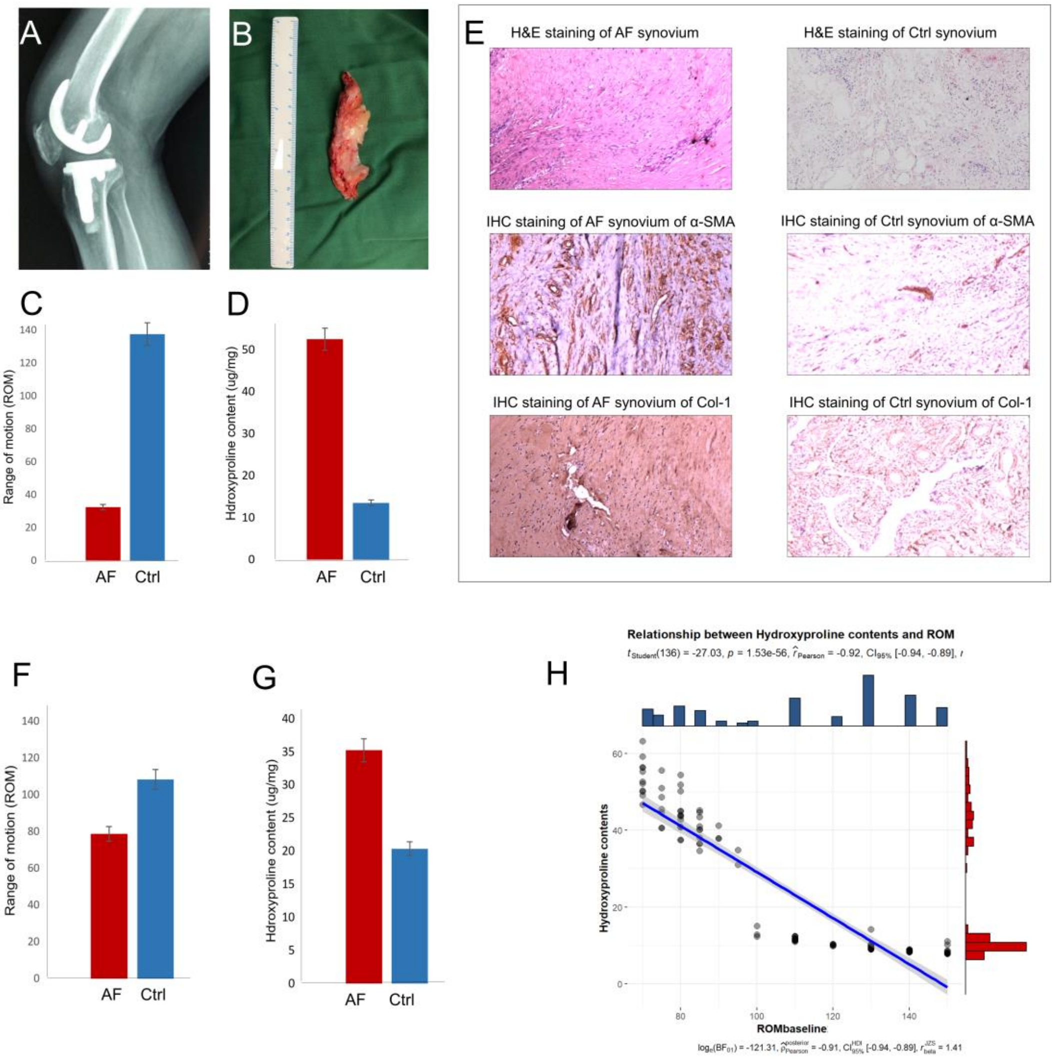

Supplementary fig. 2 Imaging and pathological analysis of arthrofibrosis patients, Related to Figure 1. **(A)** Representative X-ray image of patients' arthrofibrosis knee. **(B)** Representative gross image of arthrofibrosis synovial tissue. **(C)** Comparison of the range of motion (ROM) between scRNA-seq arthrofibrosis patients and scRNA-seq non-arthrofibrosis patients. **(D)** Comparison of the hydroxyproline contents between scRNA-seq arthrofibrosis patients and scRNA-seq non-arthrofibrosis patients. **(E)** Representative H&E staining and IHC of all arthrofibrosis patients and all non-arthrofibrosis patients. (F) Comparison of the range of motion (ROM) between all arthrofibrosis patients and all non-arthrofibrosis patients. (G) Comparison of the hydroxyproline contents between arthrofibrosis patients and non-arthrofibrosis patients. (H) Correlation between hydroxyproline contents and ROM baseline

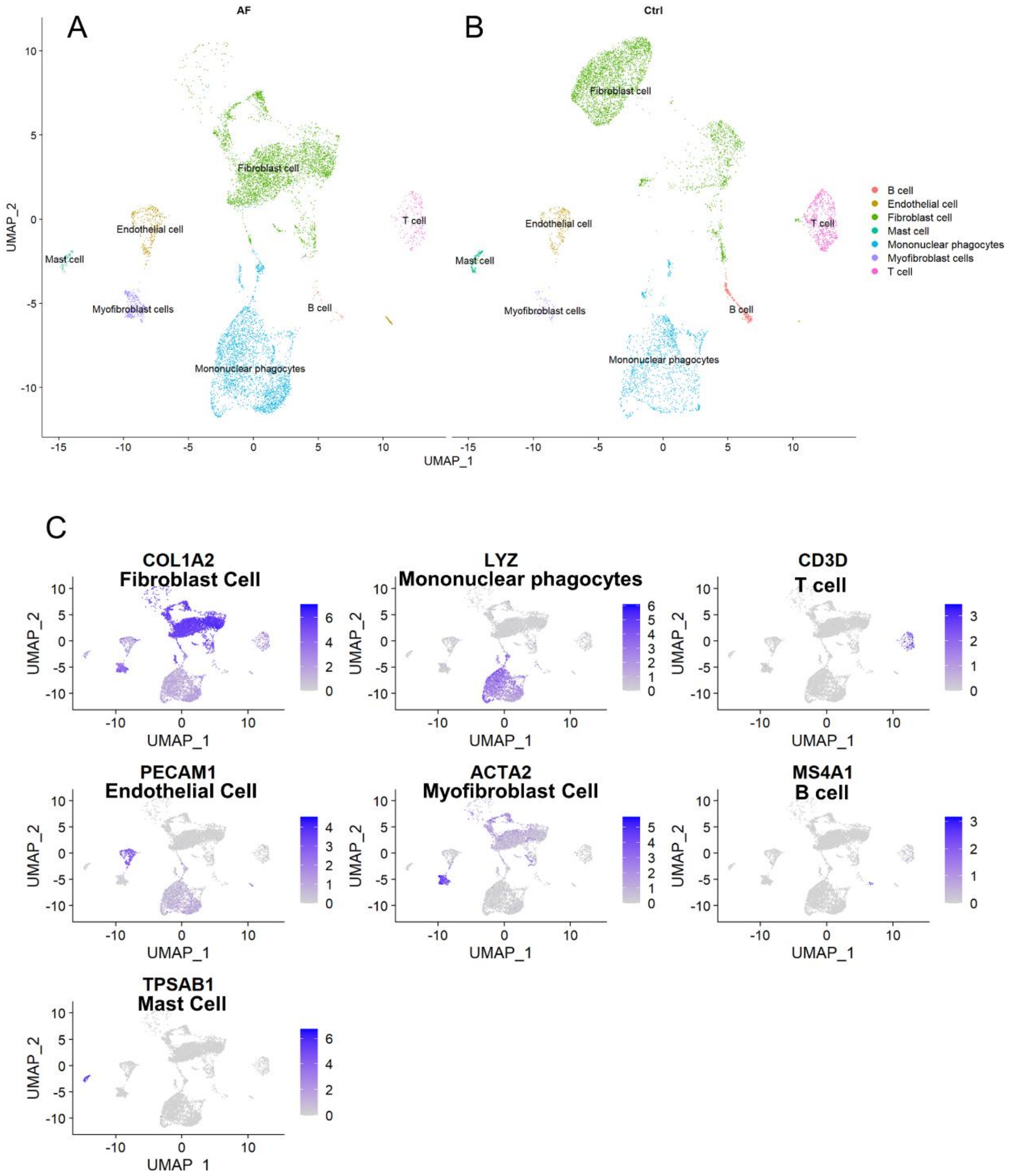

Supplementary fig. 3 Unsupervised clustering of single-cell RNA sequencing, Related to Figure 1. **(A, B)** Unsupervised clustering of single-cell RNA sequencing splitting by AF group and Non-AF group. **(C)** Feature plot of identified marker genes in each cell type.

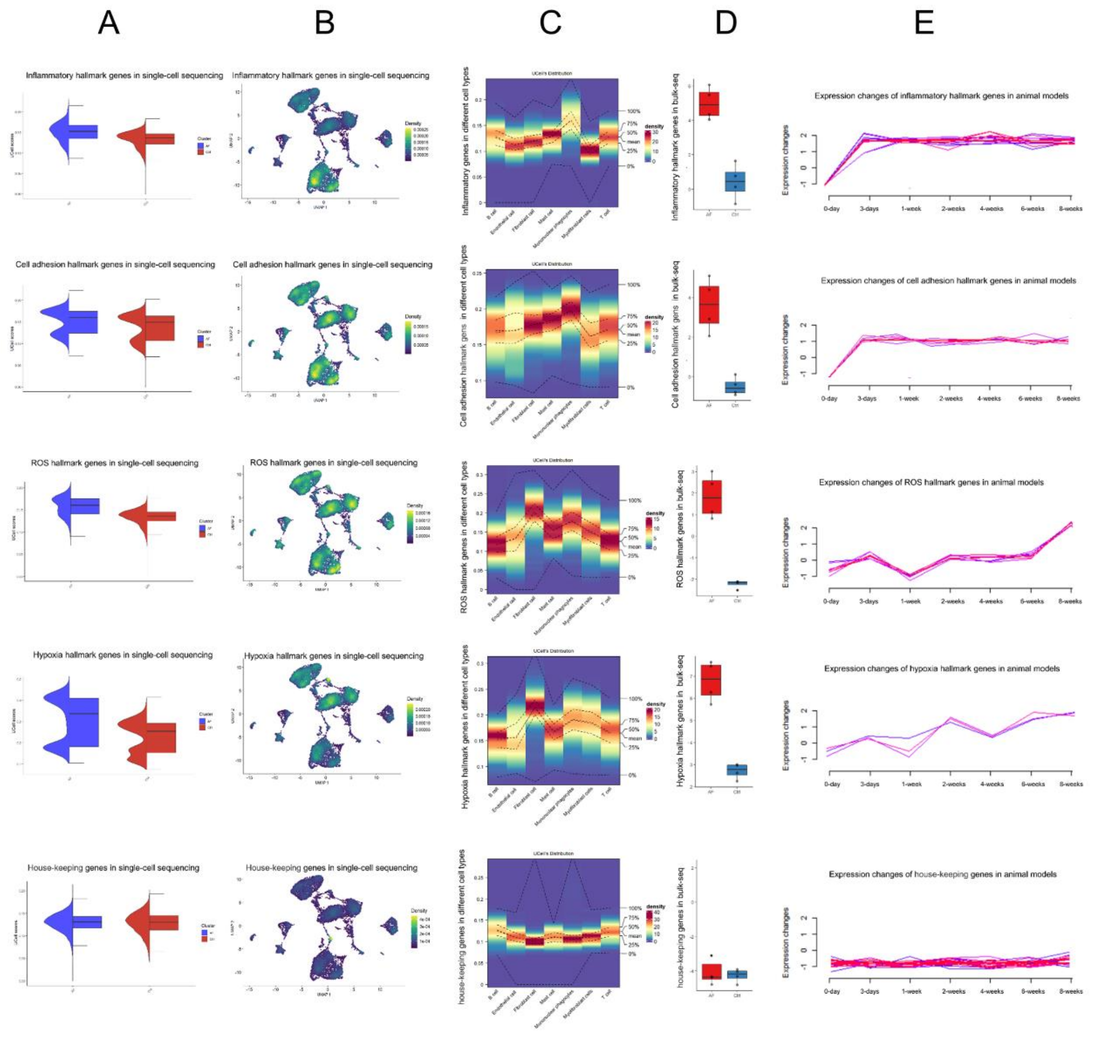

Supplementary fig. 4 Cell state analysis, Related to Figure 1. **(A-C)** Semi-Violin plot, density scatterplot, density heatmap of 5 different hallmark gene sets involving single-cell RNA sequencing data. **(D)** Box plot of the average expression level of 5 different hallmark gene sets involving human bulk RNA sequencing data. **(E)** Fold-line plot of the gene expression level of 5 different hallmark gene sets in animal model bulk RNA sequencing data.

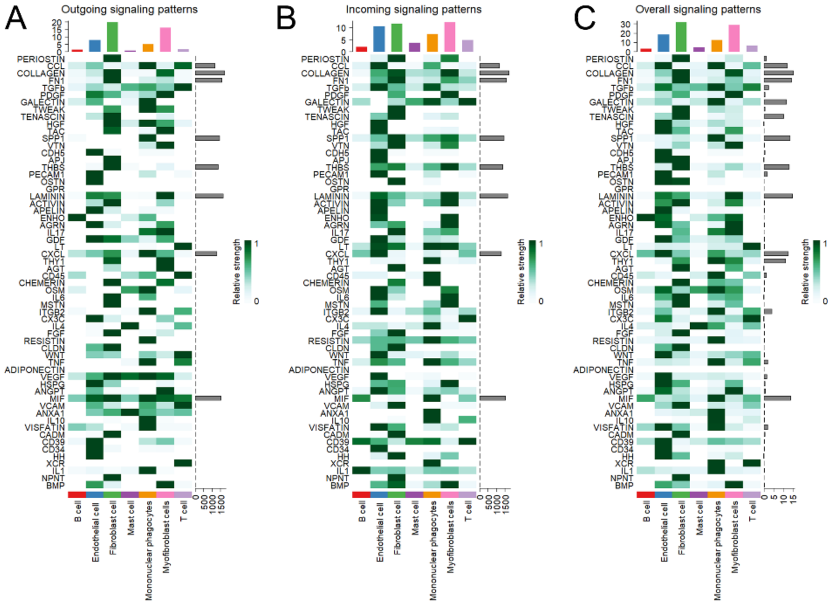

Supplementary fig. 5 All fibrosis-related cell interactions, Related to Figure 2. **(A)** Outgoing signaling patterns of all fibrosis-related cell interactions. **(B)** Incoming signaling patterns of all fibrosis-related cell interactions. **(C)** Overall signaling patterns of all fibrosis-related cell interactions.

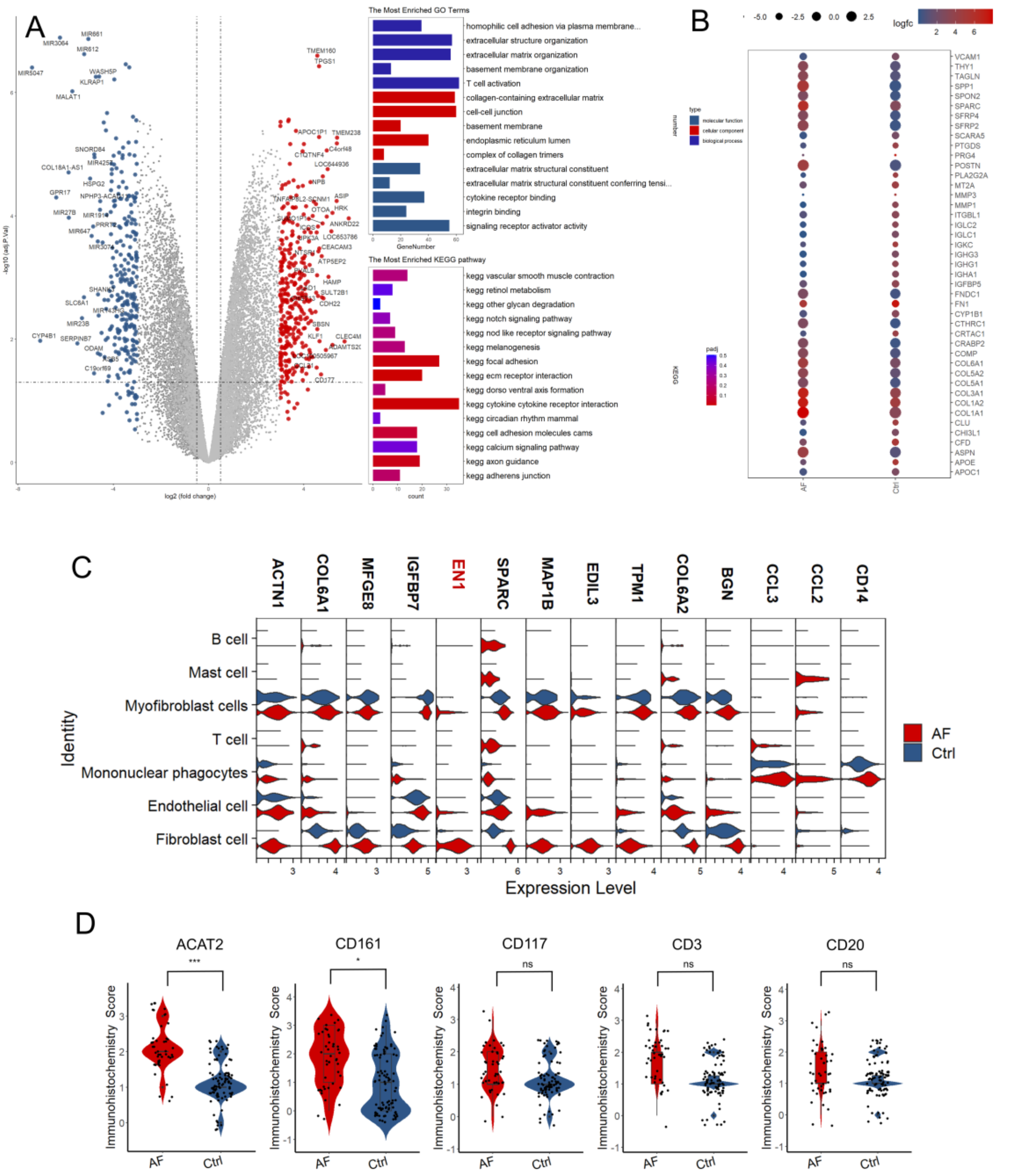

Supplementary fig. 6 Common differential genes identification, Related to Figure 3. **(A)** Volcano plot and functional enrichment analysis plot of differential expressed genes (DEG) in human bulk sequencing data. **(B)** Dot plot of differential expressed genes (DEG) in human single-cell RNA sequencing data. **(C)** Violin plot of 16 intersection genes between bulk-seq and sc-RNA seq.(D) Violin plot of IHC score evaluation of different marker genes in clinical patients cohort.

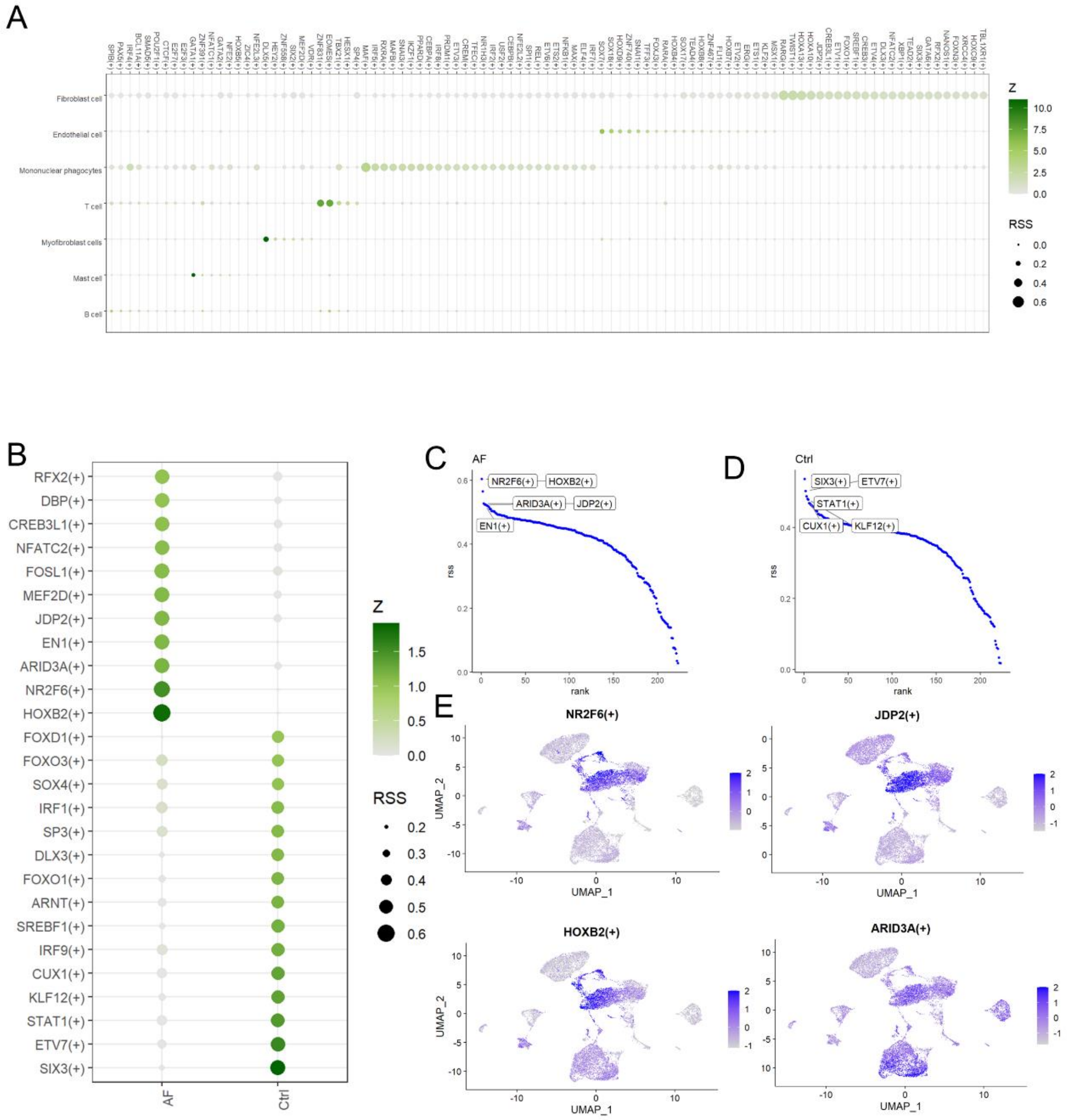

Supplementary fig. 7 Regulatory network establishment, Related to Figure 3. **(A, B)** Dot plot of significantly changing transcription factors between AF group and control group. **(C, D)** The top ranked transcription factors in AF and control group. **(E)** Feature plot of important transcription factors.

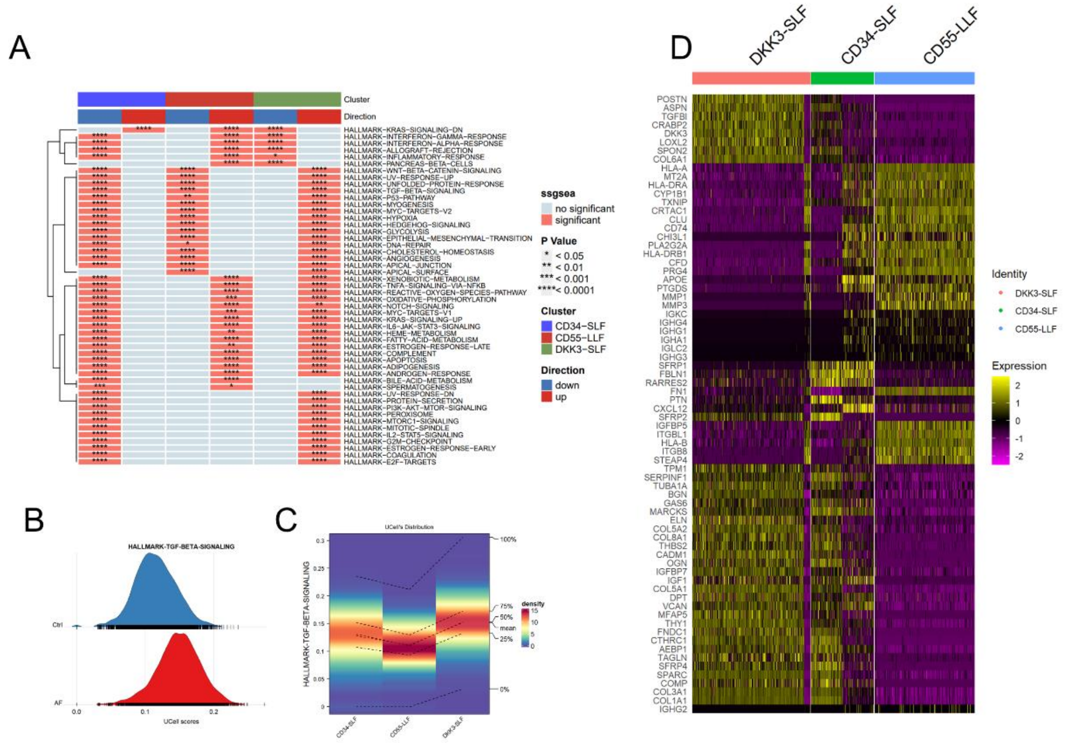

Supplementary fig. 8 Functional enrichment of fibroblast subcluster cells, Related to Figure 4. **(A)** Heatmap of significantly changed pathways of different fibroblast subclusters compared with the control group. **(B)** Comparison of TGF-b signaling pathway UCell scores between the AF group and the control group. **(C)** Density heatmap of TGF-b signaling pathway UCell scores in different fibroblast subcluster cells. **(D)** Heatmap of the top markers of fibroblast subcluster cells.

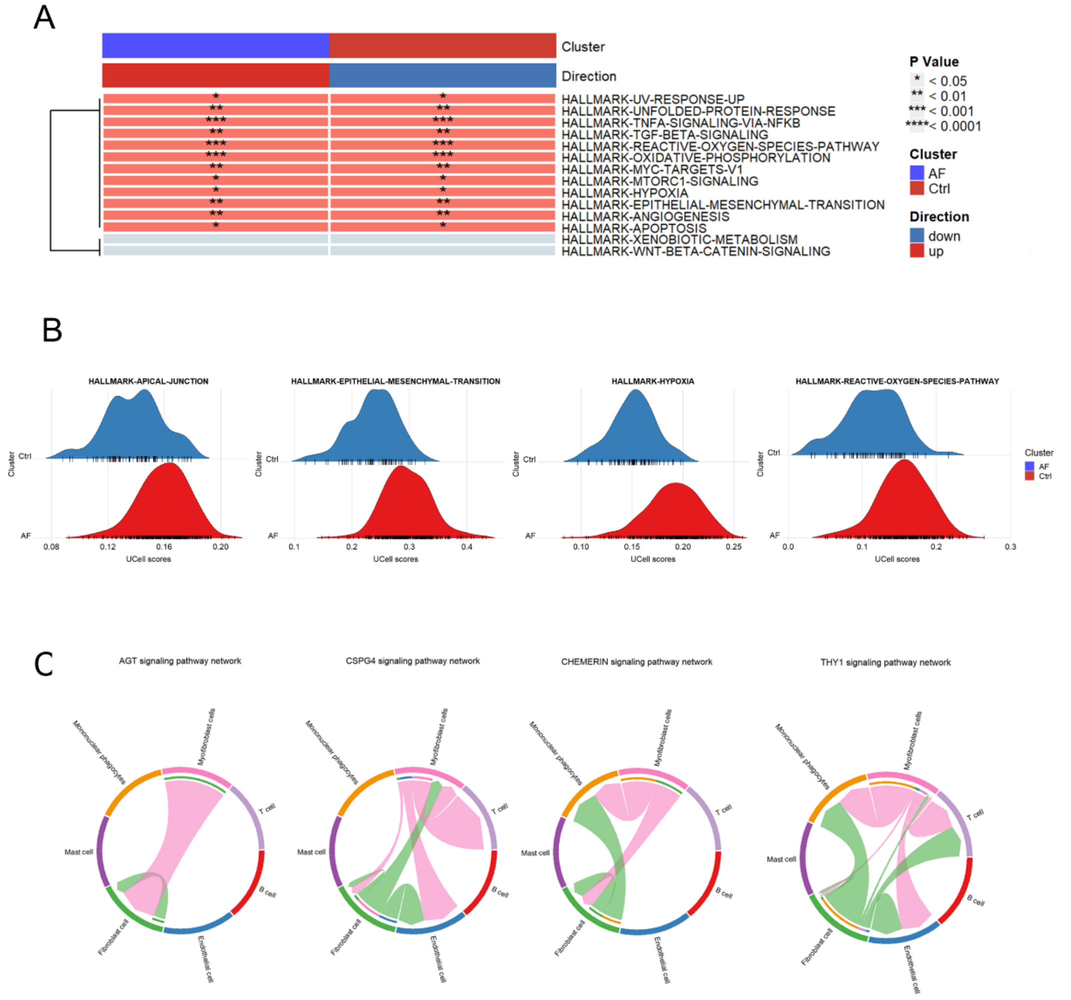

Supplementary fig. 9 Functional enrichment of myofibroblast cells, Related to Figure 5. **(A)** Heatmap of significantly changed pathways of myofibroblasts compared with the control group. **(B)** Ridge plot of different biological process comparisons. **(C)** Chord diagram of different pathway networks, including the AGT signaling pathway, CSPG4 signaling pathway, CHEMERIN signaling pathway, and THY1 signaling pathway.

Supplementary Table 1. Inclusive and exclusive criteria of the AF patients, Related to Figure 1.

Number	Inclusive criteria	Exclusive criteria
1	Persistent limitation of flexion ROM<90° at minimum 1 year follow-up	Prosthetic joint infection
2	Revision of TKA surgery for high degree of psychological strain and restriction of quality of life	Revision of TKA surgery for instability or loosening of the implantation
3	Inform consent of the patients	No obvious fibrous tissue under the microscope

Supplementary Table 2. Patient demographics for revision TKA arthrofibrosis group (AF) and revision TKA for non-arthrofibrosis group (Non-AF), Related to Figure 1.

Group	Number of patients	Mean age, years (SD)	Sex	Mean BMI, kg/m2 (SD)	Smokers	Diabetics	Mean time to RTKA, years	Mean preoperative ROM
Arthrofibrotic (AF)	47	67.01 (±12.04)	21 Male<br>26 Female	26.46 (±4.46)	17% (n =8)	11% (n = 5)	2.9 (±1.4)	78.72 (±7.41)
Non-Arthrofibrotic (Non-AF)	94	68.78 (±8.62)	43 Male<br>51 Female	23.63 (±3.61)	13% (n = 12)	10% (n = 9)	7.7 (±2.3)	108.29 (±13.64)

Supplementary Table 4. Differential expressed genes of single-cell sequencing data, Related to Figure 1

Gene	p_val	avg_log2FC	pct.1	pct.2	p_val_adj
COL11A1	0	1.307668	0.366	0.011	0
ECM1	0	1.185264	0.753	0.486	0
LMNA	0	1.334893	0.953	0.861	0
CRABP2	0	2.428509	0.589	0.116	0
ANTXR1	0	1.086281	0.549	0.269	0
COL3A1	0	3.168952	0.965	0.623	0
COL5A2	0	2.120421	0.685	0.534	0
COL8A1	0	1.758442	0.525	0.103	0
SPON2	0	2.255443	0.576	0.158	0
IGFBP7	0	1.737268	0.846	0.631	0

SPP1	0	4.989659	0.639	0.026	0
SFRP2	0	3.356719	0.588	0.024	0
PALLD	0	1.352951	0.67	0.365	0
MAP1B	0	1.524775	0.576	0.035	0
VCAN	0	1.098284	0.713	0.39	0
EDIL3	0	1.380441	0.55	0.05	0
LOX	0	1.104819	0.508	0.091	0
TGFBI	0	1.946909	0.876	0.386	0
SPARC	0	3.185952	0.949	0.696	0
FNDC1	0	2.133849	0.551	0.08	0
THBS2	0	1.670217	0.574	0.183	0
SFRP4	0	2.414373	0.567	0.059	0
AEBP1	0	1.973229	0.652	0.274	0
ELN	0	1.850391	0.53	0.24	0
COL1A2	0	2.505236	0.973	0.709	0
SERPINE1	0	1.368547	0.486	0.137	0
LRRC17	0	1.172174	0.468	0.136	0
LOXL2	0	1.648958	0.543	0.142	0
CTHRC1	0	2.419912	0.613	0.069	0
TPM2	0	1.619801	0.629	0.35	0
OGN	0	1.592139	0.51	0.095	0
ASPN	0	3.410249	0.707	0.123	0
RGS3	0	1.197038	0.565	0.321	0
CERCAM	0	1.289702	0.603	0.431	0
COL5A1	0	2.080932	0.639	0.31	0
PLAU	0	1.029324	0.721	0.358	0
ACTA2	0	1.963148	0.515	0.234	0
ADAM12	0	1.156346	0.515	0.015	0
DKK3	0	1.940963	0.605	0.112	0
SERPINH1	0	1.654009	0.703	0.523	0
CADM1	0	1.711971	0.566	0.096	0
TAGLN	0	2.427863	0.634	0.188	0
THY1	0	2.189869	0.637	0.27	0
OAF	0	1.017029	0.578	0.305	0
GAPDH	0	1.143166	0.983	0.95	0
MFAP5	0	1.732972	0.521	0.032	0
TUBA1A	0	1.60936	0.904	0.738	0
POSTN	0	3.994608	0.746	0.039	0
GAS6	0	1.124582	0.781	0.315	0
ACTN1	0	1.375429	0.792	0.213	0
TPM1	0	1.58852	0.679	0.382	0
ACAN	0	1.250825	0.412	0.007	0
TNFRSF12A	0	1.528976	0.585	0.159	0

COL1A1	0	4.723753	0.981	0.584	0
RAB31	0	1.060027	0.848	0.529	0
TPM4	0	1.054754	0.913	0.751	0
COMP	0	2.365581	0.555	0.074	0
PLAUR	0	1.031794	0.712	0.32	0
MYADM	0	1.532944	0.88	0.594	0
COL6A1	0	2.249504	0.797	0.635	0
COL6A2	0	1.621055	0.808	0.652	0
LGALS1	0	1.361238	0.973	0.876	0
CD99	0	1.064101	0.966	0.897	0
MXRA5	0	1.232221	0.561	0.329	0
BGN	0	1.703146	0.722	0.538	0
F13A1	6.55E-303	1.268606	0.278	0.042	2.40E-298
H19	2.04E-288	1.068285	0.206	0.008	7.47E-284
CCL3L1	7.17E-266	1.855139	0.51	0.223	2.62E-261
MYL9	3.70E-259	1.41655	0.668	0.557	1.36E-254
COL12A1	4.45E-228	1.113486	0.582	0.407	1.63E-223
CCL3	1.63E-204	1.404214	0.499	0.251	5.97E-200
DPT	2.30E-200	1.32448	0.578	0.397	8.43E-196
SERPINF1	3.00E-198	1.058043	0.718	0.601	1.10E-193
MFGE8	2.12E-177	1.017125	0.619	0.535	7.77E-173
CCL4L2	2.66E-176	1.263668	0.518	0.276	9.72E-172
ANGPTL2	8.39E-175	1.479685	0.615	0.532	3.07E-170
CTSK	6.55E-161	1.138376	0.644	0.603	2.40E-156
CXCL2	1.13E-134	1.234129	0.559	0.371	4.13E-130
TYROBP	1.42E-123	1.04717	0.444	0.287	5.19E-119
MMP2	4.49E-123	1.077746	0.764	0.689	1.64E-118
CCL2	2.23E-108	1.072584	0.5	0.318	8.15E-104
CXCL8	3.09E-96	1.555828	0.474	0.315	1.13E-91
CXCL3	7.79E-55	1.408955	0.476	0.371	2.85E-50
CD14	2.62E-11	1.324872	0.472	0.517	9.58E-07
